# Arthropod Distribution in a Tropical Rainforest: Tackling a Four Dimensional Puzzle

**DOI:** 10.1371/journal.pone.0144110

**Published:** 2015-12-03

**Authors:** Yves Basset, Lukas Cizek, Philippe Cuénoud, Raphael K. Didham, Vojtech Novotny, Frode Ødegaard, Tomas Roslin, Alexey K. Tishechkin, Jürgen Schmidl, Neville N. Winchester, David W. Roubik, Henri-Pierre Aberlenc, Johannes Bail, Héctor Barrios, Jonathan R. Bridle, Gabriela Castaño-Meneses, Bruno Corbara, Gianfranco Curletti, Wesley Duarte da Rocha, Domir De Bakker, Jacques H. C. Delabie, Alain Dejean, Laura L. Fagan, Andreas Floren, Roger L. Kitching, Enrique Medianero, Evandro Gama de Oliveira, Jérôme Orivel, Marc Pollet, Mathieu Rapp, Sérvio P. Ribeiro, Yves Roisin, Jesper B. Schmidt, Line Sørensen, Thomas M. Lewinsohn, Maurice Leponce

**Affiliations:** 1 Smithsonian Tropical Research Institute, STRI-Research, 080814 Panama City, Republic of Panama; 2 University of South Bohemia, Biological Faculy, 370 05 Ceske Budejovice, Czech Republic; 3 Universidad de Panamá, Maestria de Entomologia, 080814 Panama City, Republic of Panama; 4 Biology Centre of the Czech Academy of Sciences, Institute of Entomology, 370 05 Ceske Budejovice, Czech Republic; 5 Muséum d'histoire naturelle de la Ville de Genève, Département des arthropodes et d'entomologie I, 1208 Genève, Switzerland; 6 The University of Western Australia, School of Animal Biology and CSIRO Land & Water, 6009 Perth, Australia; 7 Norwegian Institute for Nature Research, Trondheim, 7485 Trondheim, Norway; 8 Swedish University of Agricultural Sciences, Department of Ecology, SE-750 07 Uppsala, Sweden; 9 National Museum of Natural History, Department of Entomology, Washington, DC 20013–7012, United States of America; 10 University of Erlangen-Nuremberg, Department of Biology, 91058 Erlangen, Germany; 11 University of Victoria, Department of Biology,Victoria, BC V8W 2Y2, Canada; 12 Cirad, Centre de Biologie pour la Gestion des populations, 34988 Montferrier-sur-Lez, France; 13 Am Ehrenbach 8, 91356 Kirchehrenbach, Germany; 14 University of Bristol, School of Biological Sciences, Bristol BS8 1TH, United Kingdom; 15 Universidad Nacional Autónoma de México, Facultad de Ciencias, 76230, Querétaro, México; 16 CNRS, UMR 6023, 63177 Aubière & Université Blaise Pascal, 63000 Clermont-Ferrand, France; 17 Museo Civico di Storia Naturale, 10022 Carmagnola, Italy; 18 Universidade Federal de Minas Gerais, Instituto de Ciências Biológicas, 31270–901 Belo Horizonte, Brazil; 19 Institut Royal des Sciences Naturelles de Belgique, Operational Directorate Natural Environment, 1000 Brussels, Belgium; 20 Centro de Pesquisas do Cacau, Convênio UESC-CEPLAC, 45600–000, Itabuna & Universidade Estadual de Santa Cruz, 45662–900 Ilhéus-Bahia, Brazil; 21 University of Toulouse III, UMR EcoFoG, 31062 Toulouse, France; 22 Western Australia Department of Agriculture and Food, Biosecurity and Regulations, 6151 Perth, Australia; 23 Universität Würzburg, Department of Animal Ecology and Tropical Biology, 97070 Würzburg, Germany; 24 Griffith University, School of Environment, Nathan QLD 4111, Australia; 25 Centro Universitário Una, Ciências Biológicas, 30180–100 Belo Horizonte, Brazil; 26 CNRS, UMR EcoFoG, 97379 Kourou, France; 27 Research Institute for Nature and Forest, Research Group Species Diversity, 1070 Brussels, Belgium; 28 Le bois Gervaz, 74440 Mieussy, France; 29 Universidade Federal de Ouro Preto, Instituto de Ciências Exatas e Biológicas, 35400–000 Ouro Preto-MG, Brazil; 30 Université Libre de Bruxelles, Evolutionary Biology and Ecology, 1050 Brussels, Belgium; 31 Vetterslev Bygade 27, 4100 Ringsted, Denmark; 32 Chemin de la Treille 7b, 1297 Founex, Switzerland; 33 University of Campinas, Departamento de Biologia Animal, 13083–870 Campinas, Brazil; University of Colorado, UNITED STATES

## Abstract

Quantifying the spatio-temporal distribution of arthropods in tropical rainforests represents a first step towards scrutinizing the global distribution of biodiversity on Earth. To date most studies have focused on narrow taxonomic groups or lack a design that allows partitioning of the components of diversity. Here, we consider an exceptionally large dataset (113,952 individuals representing 5,858 species), obtained from the San Lorenzo forest in Panama, where the phylogenetic breadth of arthropod taxa was surveyed using 14 protocols targeting the soil, litter, understory, lower and upper canopy habitats, replicated across seasons in 2003 and 2004. This dataset is used to explore the relative influence of horizontal, vertical and seasonal drivers of arthropod distribution in this forest. We considered arthropod abundance, observed and estimated species richness, additive decomposition of species richness, multiplicative partitioning of species diversity, variation in species composition, species turnover and guild structure as components of diversity. At the scale of our study (2km of distance, 40m in height and 400 days), the effects related to the vertical and seasonal dimensions were most important. Most adult arthropods were collected from the soil/litter or the upper canopy and species richness was highest in the canopy. We compared the distribution of arthropods and trees within our study system. Effects related to the seasonal dimension were stronger for arthropods than for trees. We conclude that: (1) models of beta diversity developed for tropical trees are unlikely to be applicable to tropical arthropods; (2) it is imperative that estimates of global biodiversity derived from mass collecting of arthropods in tropical rainforests embrace the strong vertical and seasonal partitioning observed here; and (3) given the high species turnover observed between seasons, global climate change may have severe consequences for rainforest arthropods.

## Introduction

The majority of terrestrial eukaryote diversity on Earth is represented by arthropods in tropical rainforests. The diversity of arthropod feeding guilds and functional niches observed in tropical rainforests is also unparalleled [1,2]. Comparatively little is known, however, about the factors driving high spatio-temporal variation in this local diversity among its component dimensions of space and time. We propose that (1) quantifying the relative influence of local factors affecting the present day distribution of arthropods is a necessary first step towards scrutinizing the global distribution of biodiversity on Earth, and (2) assessing these factors for different taxa and functional groups is crucial for understanding biodiversity and for developing sound strategies for arthropod conservation in tropical rainforests. Here, the mere documentation of spatio-temporal patterns and of how they vary among taxa represent an important first step in itself.

Ecologists are often concerned with quantifying animal or plant distribution with regard to historical factors [3], latitudinal or altitudinal gradients [4], geographic distance [5] or seasonality [6]. All these dimensions of species diversity depend on scale [7,8] and the appropriate scale for examination may also depend on the ecosystem and the organisms considered. For example, many ecological models include the pattern of decreasing community similarity with geographical distance [5,9]. It is thought that the distance decay of similarity can be attributed to three potential mechanisms: niche-based processes, spatial configuration dictating the dispersal rate of organisms, and neutral processes such as random dispersal or speciation [9,10]. Although these are often treated as branching alternatives, niche-based explanations are (in principle) not necessarily incompatible with dispersal limitation in an ecologically neutral habitat [11]. We would further suggest here that these concepts might apply diversely to different taxa. Even though not as widely recognized, the distance decay of similarity can also be applied in temporal studies, where similarity can be plotted against the temporal distance between observations [12].

With respect to tropical arthropod communities, the scale of a whole rainforest, or a large portion of say several square kilometers, surpasses the trivial dispersal distance of most species by several orders of magnitude. At these scales, it is realistic to assess both the horizontal and vertical spatial turnover among local communities, and whether or not these are determined by differences in habitat structure or physical factors, over and above random dispersal dynamics on an ecological time-scale. Understanding the drivers of the spatio-temporal organization of communities at the scale of the forest is essential to understanding and conserving tropical arthropod biodiversity [13]. If beta-diversity scaling relationships differ among disparate organisms, then conservation strategies aimed at preventing the loss of species will have to consider the requirements of multiple taxa at multiple spatial scales [13]. To date, most studies have focused on a few assemblages only (e.g. butterflies, beetles: [14–17]) and have rarely been designed to allow the explicit partitioning of components of arthropod diversity. Here, we consider a uniquely large dataset for the San Lorenzo forest in Panama. Within this forest, we surveyed the full phylogenetic breadth of arthropod taxa using 14 structured protocols targeting microhabitats from the soil to the upper canopy, and replicated across seasons in 2003 and 2004. We use this dataset to explore the relative contributions of horizontal, vertical and seasonal variation to the total distribution in space and time of arthropods in this forest.

In lowland tropical rainforests, one of the obvious spatial gradients of species change (hereafter referred to as ‘dimension’, so as not to assume the existence of an autocorrelated gradient per se) is related to geographic distance, as tropical rainforests are notoriously heterogeneous environments [18]. Faunal changes in this horizontal dimension may be driven by multiple factors, including forest dynamics [19], the presence of host-plants and their relatives for herbivores [20], soil properties affecting the quality of host-plants for herbivores [21], distance from forest edge [22], or even neutral processes that incorporate distance-decay of dispersal [18]. Since geographic distance is two-dimensional, the spatio-temporal distribution of arthropods in a rainforest is actually described by four dimensions (hence the title of our contribution).

A seasonal dimension may result from temporal heterogeneity in the biodiversity of different habitats, reflecting varying availability of resources, or microclimates [23]. It has long been known that faunal changes induced by seasonality can be significant in “aseasonal” tropical climates [24]. Moreover, other biotic processes, such as resource competition and predation/prey dynamics, also influence this seasonal dimension, particularly in tropical rainforests [25]. We thus use the term “seasonal dimension” to distinguish these effects from those related to diel activity and inter-annual changes, even though these may also be important drivers of variation in biodiversity. Plant growth is shaped by competition for light, promoting floristic changes in the vertical dimension, from forest floor to canopy [26]. Faunal changes, promoted by various mechanisms, are also significant within this dimension [15]. In forests, the vertical dimension encompasses habitats from the subsoil to the upper canopy.

A priori, we expect that in tall closed tropical rainforests plant turnover may be substantial between the understory and the upper canopy (see below for definition of these terms). Hence we conjecture that at the scale of the whole forest stand (or slightly smaller scales) changes in arthropod species along the vertical dimension are substantial, and more important than corresponding changes observed in the horizontal and temporal dimensions. We also expect differences between short-lived (arthropods) and long-lived (trees) organisms with the temporal dimension being more significant for the former within the seasonal dimension. In this context, we ask the following specific questions:

1. What is the relative contribution of horizontal, vertical and seasonal dimensions to the variation of arthropod biodiversity in a closed-canopy tropical rainforest? In which dimension(s) are arthropod assemblages most similar? We investigate these two questions by quantifying several diversity components, including arthropod abundance, observed and estimated species richness, additive decomposition of species richness, multiplicative partitioning of species diversity, variation in species composition, species turnover and guild structure.
2. Do patterns of distribution in the horizontal, vertical and seasonal dimensions differ among arthropod feeding guilds?
3. If analyzed at the same scale (i.e., each dimension bounded by its maximum state as studied here), are distributional patterns in the horizontal, vertical and seasonal dimensions different for arthropods and trees in this forest? In particular, are models of beta diversity developed for tropical trees [27] relevant to tropical arthropods, which represent the majority of biodiversity in tropical forests?

## Materials and Methods

### Study sites

**Fig 1:**
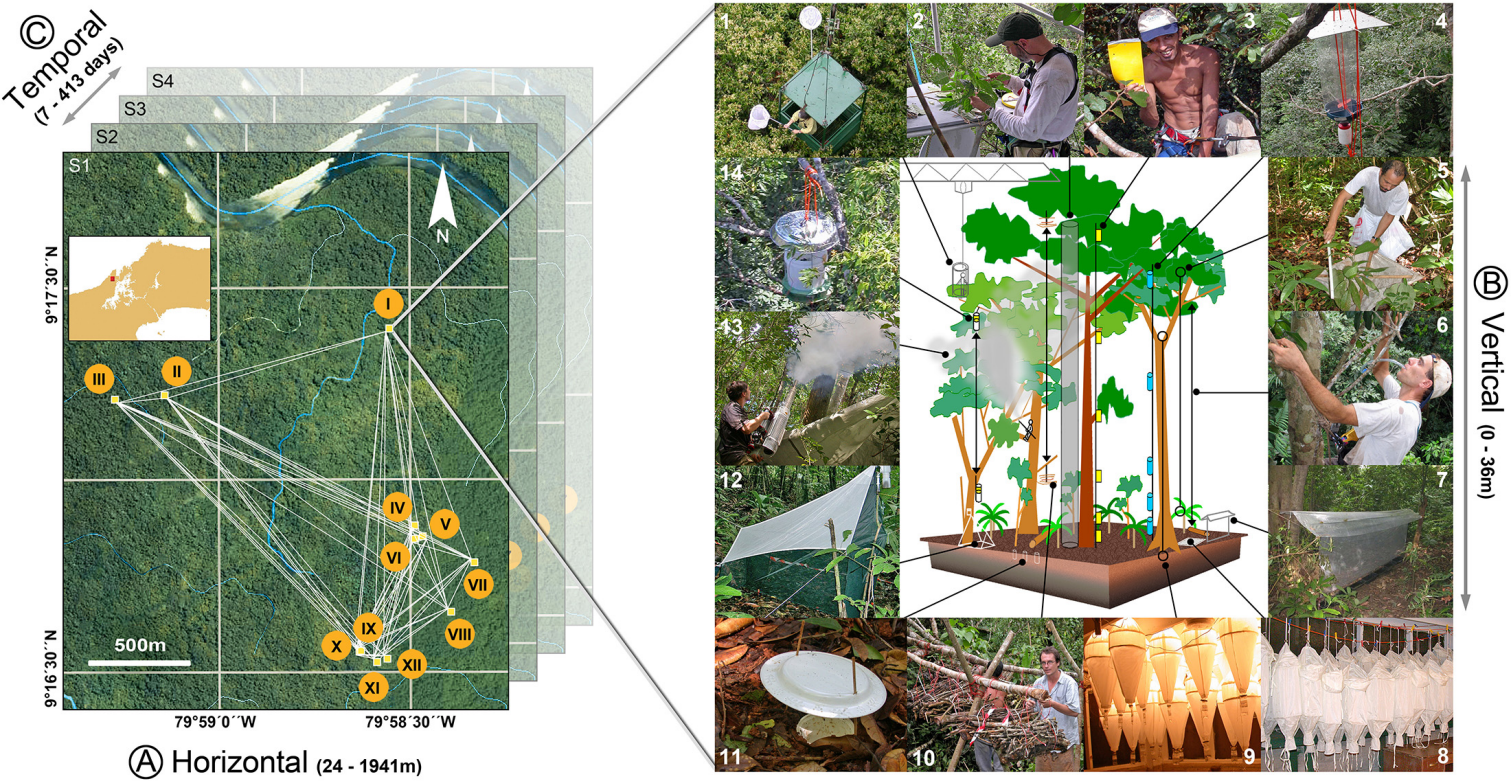
Overview of the sampling design. Three major spatio-temporal dimensions were considered for arthropods: spatial turnover or difference in species composition measured (A) horizontally (among sites, all less than 2km apart), (B) vertically from -5cm to 36m above ground, and (C) temporally, among sampling intervals repeated within a period of 413 days. (A) Twelve 20x20m sites (I-XII) were surveyed for plants and arthropods, from the ground to the upper canopy. (B) Arthopods were surveyed using 14 different protocols from ground to the upper canopy: (1) baits and netting; (2) gall sampling within the volumetric space of a vertical cylinder; (3) sticky traps; (4) aerial composite flight-interception traps; (5) beating of vegetation and dead branches; (6) hand collecting for ants and termites; (7) ground flight-interception traps; (8) collection of the leaf-litter fauna and extraction with a mini-Winkler apparatus; (9) collection of ground and suspended soils, extraction with Berlese-Tullgren apparatus; (10) wood rearing; (11) pitfall traps; (12) ground Malaise traps; (13) canopy fogging; (14) light traps. (C) After an initial sampling period of 6 weeks during the late wet season (September-October 2003, hereafter Survey S1), field work was replicated during three similar sampling periods targeting the dry, early wet and late wet seasons (Survey S2: February-March 2004, Survey S3: May-June 2004, Survey S4: October-November 2004). Photos by JS (1), SR (2), ML (3,5,8,9,11,13), NW (4), N. Baiben (6), C.E. Carlton (7), M. Janda & J. Patera (10), RLK (14), S. Pinzon (12).

Field sampling was performed in the San Lorenzo Protected Area in Panama (9°16’N, 79°58’W, elevation ca 130 m a.s.l.). Annual rainfall in this lowland wet forest averages 3,139 mm and annual average air temperature is 26.0°C. Fifty percent of this area is a contiguous evergreen seasonal mixed forest (6,000ha; see [28] for a detailed account) within which our study area was located. Twelve 20 x 20 m sites (coded as B1, B2, C1, C2, C3, F1, F2, F3, I1, R1, R2 and R3), all less than 2 km apart, were surveyed for plants and arthropods, from the ground to the upper canopy. Plots of 400m^2^ have been shown to be adequate to evaluate tree diversity [29] and arthropod diversity [30] in tropical rainforests. At all sites, plants > 1cm diameter at breast height (DBH) were tagged and identified before arthropod collections began. Sampling in the upper canopy deployed a combination of fogging, single-rope climbing techniques and several devices such as a canopy crane, canopy raft, canopy bubble and tree-platform. The location, description, vegetation characteristics of all sites and logistics of accessing the upper canopy are detailed in [30] (and see also Fig 1).

### Arthropod collecting and processing

Arthropods were surveyed using 14 different protocols targeting the soil, litter, understory, mid-canopy and upper canopy habitats, replicated across seasons in 2003 and 2004. Hence our protocols ensured that most of the vertical profile of the forest was surveyed. The soil was surveyed to a depth of 5cm. For simplicity, samples of soil and litter were jointly regarded as “litter”. Understory samples were collected 0 to 3m above the forest floor, lower canopy (hereafter “canopy” for sake of brevity) samples were collected from 3 to 35m above the ground, and upper canopy samples from heights above 35m. This last habitat may be defined as the canopy surface (the interface between the uppermost layer of leaves and the atmosphere) and the volume immediately below (a few meters; [15]). The above terms do not necessarily mean that the forest is stratified: they are simply convenient to describe the vertical location of samples.

To analyse the samples, we established a consortium of 102 researchers with expertise encompassing the full breadth of phylogenies and feeding modes present among arthropods. This consortium invested a total of 24,354 trap- (or person-) days sampling the San Lorenzo forest using structured protocols [1]. These protocols included: Winkler sifting; Berlese-Tullgren extraction; hand-collecting of galls and social insects; fogging; beating; wood-rearing; baits and various types of traps such as pitfall, small and large flight-interception, sticky, light, and Malaise traps. All of the protocols and their characteristics are detailed in [30] (and see Fig 1). After an initial sampling period of 6 weeks during the late wet season (September-October 2003, hereafter Survey S1), we replicated field work during three similar sampling periods targeting the dry, early wet and late wet seasons (Survey S2: February-March 2004, Survey S3: May-June 2004, Survey S4: October-November 2004). During this extended sampling period, flight-interception, sticky and Malaise traps were run for extended periods (flight-interception traps continuously from October 2003 to October 2004). Temporal replicates thus ensured that arthropod seasonality was accounted for. No endangered species were collected or disturbed as part of the study. Fig 1 provides a schematic drawing of the full sampling design, with explicit scales and dimensions.

Focal arthropods were sorted to named species or morphospecies by taxonomists and assigned to the following arthropod guilds: ants, phytophages, fungivores, predators, parasitoids and scavengers (see S1 Text for further details about taxonomic sorting and guild assignment). The arthropod data (as of 10 May 2012) have been deposited in the Dryad repository: http://datadryad.org/review?doi=doi:10.5061/dryad.5hn8n. Field permits were granted by the Autoridad Nacional del Ambiente (ANAM).

### Arthropod data and overall rationale of statistical analyses

We considered three major spatio-temporal dimensions: spatial turnover or difference in species composition (a) among sites (horizontal dimension), (b) within the vertical dimension (vertical dimension), and (c) temporal variation among repeated sampling intervals through time (seasonal dimension). We evaluated the effects of horizontal, vertical and seasonal dimensions for four main categories of arthropod variables: (1) abundance, (2) observed and estimated species richness, (3) species composition and faunal similarity and (4) guild structure. Because of the great number of protocols used at the different sites, within different habitats and surveys, a full factorial design (sites x habitats x surveys) allowing the partitioning of insect variables among the three dimensions, was not always possible. In addition, some protocols were only valid for particular habitats (e.g., Winkler extractions targeting the litter fauna). There were also obvious physical limitations as, for example, bulky flight-interception and light traps could rarely be placed in the upper canopy, as opposed to small and light sticky traps. When partitioning the insect variance in the horizontal, vertical and seasonal dimensions, we opted to include the datasets best balanced in the dimension of relevance for the analysis, as explained below and in S1 Text. We also restricted our data to protocols that targeted multiple taxa (i.e., we excluded single taxon bee and termite protocols, [30]). This limited our analyses to the 10 following protocols, listed in order of decreasing number of individuals collected: Berlese-Tullgren, flight-intercept trap (FIT), ground flight-intercept trap, light trap, fogging, Malaise trap, Winkler, sticky trap, pitfall trap and beating.

### Arthropod abundance and species richness

For analyses of arthropod abundance and species richness we restricted the dataset to species that were collected with at least a probability of being present as a single individual at all study sites (i.e., minimal number of individuals collected ≥ 12). This entire dataset so defined included 98,793 individuals representing 1,041 species distributed among 12 sites, four habitats along vertical gradients, and four seasonal surveys, with more specific subsets used in given analyses (see below and S1 Text). To evaluate the effect of horizontal, vertical and seasonal dimensions on arthropod abundance and species richness we used Kruskal-Wallis tests, considering protocols that maximized replicates among sites, habitats or surveys, or were more comprehensive (e.g., fogging for studying the variance among sites). For each of these analyses, we calculated an effect size based on the highest and lowest mean of samples. We also performed mixed-effects ANOVAs examining the relative influence of sites, habitats and surveys with much smaller data sets appropriate to this end. In a similar fashion, we ran all of these analyses for the abundance of the main arthropod guilds collected in samples. Description of supplementary analyses are provided in S1 Text.

### Partitioning of arthropod species richness and diversity

For analyses other than those related to abundance and species richness, we used, as far as possible, the full dataset that included 113,952 individuals representing 5,858 species. There are currently two main frameworks, additive and multiplicative, to partition species richness or diversity, and both are complementary for the interpretation of a robust estimation of biodiversity [31]. Following Marcon et al. [31], we call them the additive decomposition of species richness and multiplicative partitioning of species diversity, respectively. The first analysis refers more particularly to changes in species richness irrespective of the relative abundance of species, whereas the second one takes into account the relative commonness and rarity of species, and further minimizes the influence of sample size [32]. Recent debates about additive vs. multiplicative diversity partitioning have emphasized questions about the additive partitioning of species diversity but not that of species richness [33]. As a straightforward analysis, we used the following method for the additive decomposition of species richness:

γ=α+β

where γ is the total species richness in the study system, α is the average species richness within samples and β is the average difference in species richness among samples [34,35]. We further partitioned β among temporal, horizontal and vertical components, such that

γ=α+βT+βH+βV

where α is the mean number of species collected per site, habitat and survey (four sites, C1, C2, C3, I1; three habitats, litter, understory and canopy; four surveys, S1-S4; 48 pooled samples); βT is the total number of species collected over the 4 surveys for each spatial combination of horizontal and vertical samples minus the mean number of species collected for that spatial component (12 samples); βH is the total number of species collected over the four surveys for each site minus the mean number of species collected for that site (four samples); and βV is the total number of species in the study system minus the mean number of species collected at each site. We partitioned species richness for different data sets, including all species collected using the 10 principal protocols, all species collected with FITs, the estimated number of species present in the study system collected either with the 10 protocols or with FITs, common and rare species, and arthropod guilds. The Chao2 estimator was used to estimate species richness and was calculated using EstimateS 8.20 [36]. Multiplicative partitioning of species diversity was calculated with Hurlbert’s effective number of species and as [32,33]:

γ=αxβTxβHxβV

Methods are detailed in S1 Text, as well as other aspects related to the partitioning of arthropod species richness and diversity.

### Variation in arthropod species composition and species turnover

Concepts and analyses related to beta diversity are numerous and often conflict in subtle ways ([37]; see S1 Text). Using the terminology of Anderson et al. [37], we are interested in investigating three aspects: (a) the relative partitioning of variation in community structure in response to its horizontal, vertical and temporal dimensions; (b) the rate of turnover in community structure along these three dimensions (therefore also testing whether strict gradients exist in these dimensions); and (c) the relative magnitude of turnover along particular gradients (if they exist) for different arthropod guilds. To investigate (a) we used canonical variation partitioning [38–40], for which we provide details in S1 Text.

To estimate the rate of turnover in community composition in the horizontal dimension we calculated faunal similarity between samples obtained at different sites and plotted pair-wise similarity as a function of the distance between sites. Likewise, to evaluate the rate of turnover in the vertical dimension we plotted pair-wise similarity of samples obtained at different heights against differences in height. To estimate seasonal turnover, we plotted pair-wise similarity of samples obtained during a whole week against the difference in time expressed in days. Further details are provided in S1 Text. We contrasted, in a similar fashion, faunal similarity for each arthropod guild along the horizontal, vertical and seasonal dimensions.

### Explanatory variables

Wherever possible, we quantified horizontal, vertical and seasonal gradients of arthropod distribution in relation to potential drivers of variation, rather than simply using arbitrary spatial categories. Arguably, the most important of these environmental variables may include: differences in floristic composition, tree basal area, or spatial heterogeneity in vegetation structure among sites (for horizontal gradients); light, canopy openness or leaf area index (for vertical gradients); and rainfall, temperature or tree phenology (for seasonal gradients). Measurements of all of these variables are detailed in S1 Text.

### Comparison of arthropod vs. tree distribution in the San Lorenzo forest

We compared arthropod vs. tree distribution in the horizontal, vertical and seasonal dimensions following the preceding analytical procedures. Our sampling protocols targeted adult arthropods. Typically, arthropods have short-lived adults that can reproduce immediately but for a short period. Our plant data, derived from the botanical plots at San Lorenzo, are limited to trees. Thus, in the context of this study, an adult arthropod is analogous to a mature tree in a reproductive state. Our various comparisons between trees and arthropods are based on this analogy and logic. For tree abundance, we considered the total number of stems recorded per site in the horizontal dimension, and the number of seedlings, saplings and trees per habitat in the vertical dimension (S1 Text). In the seasonal dimension, we considered litterfall data for flowers only (S1 Text), to quantify the availability of reproductive units. We compared effect sizes for arthropods and trees in the horizontal, vertical and seasonal dimensions. Additive decomposition of species richness and multiplicative partitioning of species diversity was performed with data for flowering trees during surveys 1–4. Variation partitioning was performed on these data in a fashion similar to that for arthropods, with the exception that seasonal variables included total rainfall, average temperature, wind speed and radiation during Surveys 1–4. Floristic turnover was estimated as for arthropods, with either all tree species occurring within study sites, or with only tree species that were flowering during Surveys 1–4.

## Results

### Arthropod abundance

**Fig 2:**
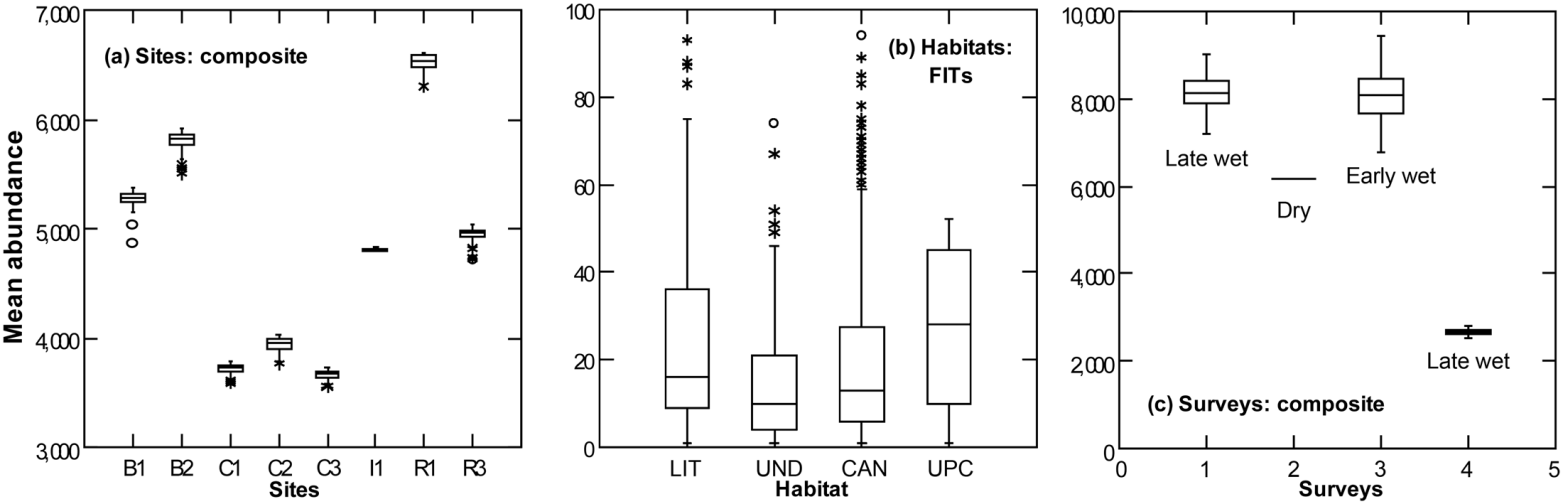
Representative box-plots of arthropod abundance across (a) sites, (b) habitats and (c) surveys. See Table 1 and methods for details about data sets.

**Fig 3:**
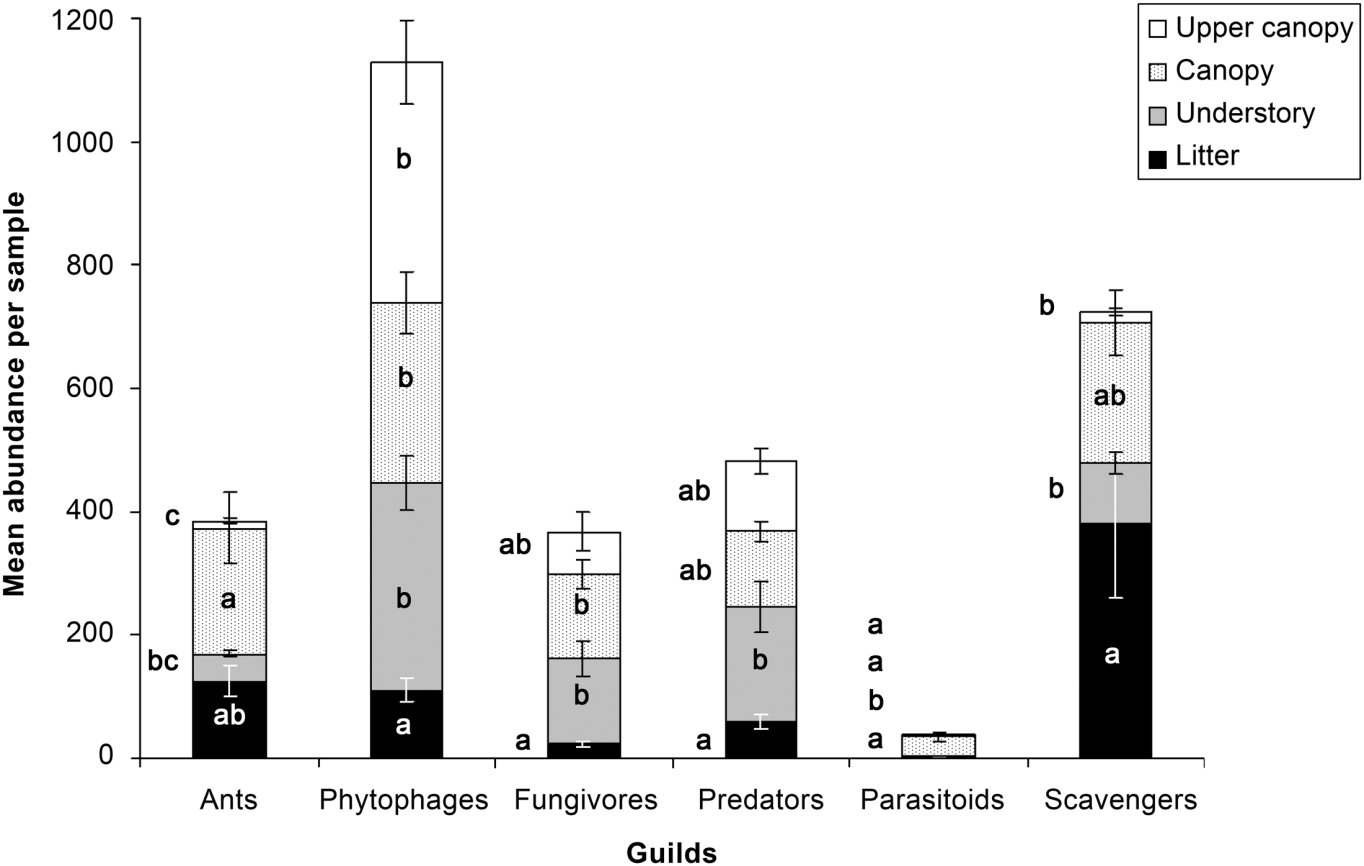
Mean (± s.e.) abundance per sample, detailed per arthropod guild and habitats (black bars = litter, grey bars = understory, stippled bars = canopy, white bars = upper canopy). ANOVAs comparing mean abundance per habitat within guilds are all significant with at least p<0.01. For each guild, different letters denote significantly different means (Tukey tests, p<0.05).

Arthropod abundance varied significantly among different surveys, but less so among sites or habitats (Table 1 & Fig 2). Arthropod abundance in sticky traps correlated with canopy openness (S1 Fig). Although the effect sizes were generally greater for seasonal comparisons (Table 1 and S1 Table, Fig 2), there was no significant difference among the effect sizes reported in Table 1 when grouped by factors (sites, habitats, surveys; Kruskall-Wallis test, W = 3.8, p = 0.149). For all arthropod guilds, with the exception of ants, mean abundance per sample was not significantly different among sites (S2 Fig). In contrast, there were sharp differences in the mean abundance of arthropod guilds among habitats (Fig 3; average ES = 0.763). For instance, scavengers were significantly more abundant in the litter, ants were significantly more abundant in the canopy, phytophages were significantly less abundant in the litter, and other guilds were more evenly distributed among habitats. The abundance of all guilds was also significantly different between surveys, often with their highest abundances during Survey 1 (S3 Fig; average ES = 0.733). Supplementary results for arthropod abundance and other arthropod variables are detailed in S2 Text.

**Table 1:** Results of Kruskall-Wallis tests comparing arthropod abundance among sites, habitats and surveys. Too few samples were available for a composite analysis of habitats.

Analysis *	No. traps /samples	No. ind.	W / *p*	Effect size	Remarks: No. sites; habitats; surveys; no. of samples or protocol considered (no. of samples) *
Sites: STI	630	1956	57.4 / <0.001	0.397	9; 4; 1; 41–92 traps
Sites: FOG	48	10777	12.8 / 0.078	-	8; 2; 1; 6 samples
Sites: FIT	275	7077	44.1 / <0.001	0.389	5; 4; 1; 53–57 samples
Sites: composite	1314	40771	779.3 / <0.001	0.280	8; 4; 1; BEA(20), BER(47), LIT(6), PIT(4), STI(41)
Habitats: STI	1150	3683	219.3 / < 0.001	0.484	9; 4; 4; 34–598 traps per habitat
Habitats: FIT	814	18092	19.5 / < 0.001	0.298	5; 4; 4; 6–535 samples per habitat
Surveys: FIT	814	18092	198.3 / < 0.001	0.641	5; 4; 4; 144–275 samples per survey
Surveys: LIT	96	14549	5.8 / 0.124	-	8; 2; 4; 12–48 samples per survey
Surveys: PIT	193	1288	23.4 / < 0.001	0.489	8; 1; 4; 27–95 samples per survey
Surveys: composite	403	39346	342.1 / < 0.001	0.507	9; 4; 4; FIT(12**), LIT(12), PIT(27), STI(8**)

* Codes of protocols: BEA = Beating, BER = Berlese-Tullgren, FITs = Flight-intercept traps, FOG = Fogging, LITs = Light traps, PITs = Pitfall traps, STIs = Sticky traps

** pooled by vertical transect at each site.

### Arthropod species richness and diversity

Species accumulation curves differed most strongly between vertical habitats (S4 Fig). Arthropod species richness, either interpolated as the median number of species collected per sample or extrapolated species richness (Chao2), followed the trends for arthropod abundance, with some slight differences. The effect of survey on species richness was significant and strong, whereas the effects of site and habitat were weaker (S2 and S3 Tables & S5 Fig). Measured effect sizes for each factor were often larger for the estimated number of species (Chao2) compared with the median number of species collected per sample.

**Fig 4:**
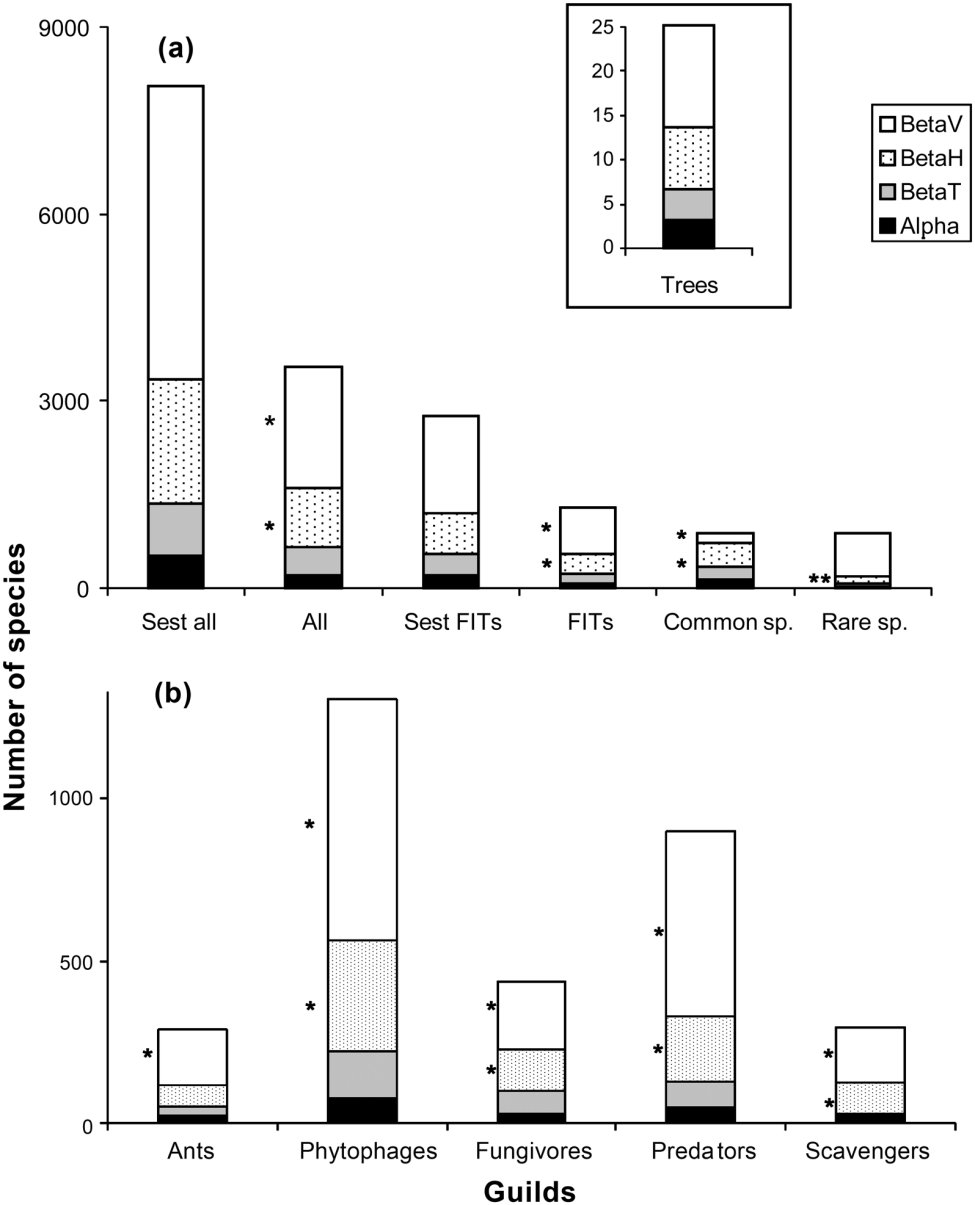
Additive decomposition of species richness for (a) major data sets and (b) arthropod guilds. (a) Major data sets (estimated species richness with ten protocols, observed species richness with ten protocols, estimated species richness with FITs, observed species richness with FITs, 885 common and rare species, inset: flowering trees (note the different scale). (b) Arthropod guilds (observed species richness with ten protocols). Species richness is partitioned among alpha (black bars), betaT (grey bars), betaH (stippled bars) and betaV (white bars). * indicates that parameters are significantly different from zero. Randomization tests were not performed with estimated species richness.

Additive decomposition of species richness indicated in general that vertical turnover (βV) > horizontal turnover (βH) > temporal turnover (βT) and this was consistent across arthropod guilds (Fig 4). The only exception to this pattern was a high horizontal turnover for common species as compared with a high vertical turnover for rare species (Chi-square = 691.9, p < 0.001). For the total number of species estimated within the study system, the proportion of change in species explained by α was 6.5%, by βT 10.4%, by βH 24.6% and by βV 58.5% (Fig 4a). Vertical turnover was higher for the guilds of phytophages and predators, as compared with ants, fungivores and scavengers (G-test = 73.09, p < 0.001; Fig 4b).

Multiplicative partitioning of species diversity indicated different patterns. In general, the effective number of fully differentiated communities accounted for by βT or βH was much higher than that accounted for βV (S6 Fig). Multiplicative βT was particularly important for all arthropods, common species and scavengers, whereas multiplicative βH was important for arthropods collected in FITs, phytophages, fungivores and predators. All multiplicative components of beta diversity were equally important for ants. For the total number of species estimated within the study system, the effective number of fully differentiated communities accounted for by βT, βH and βV was 70.0%, 2.7% and 2.4%, respectively, of the total multiplicative components of beta diversity (S6 Fig).

### Variation in species composition and species turnover

**Fig 5:**
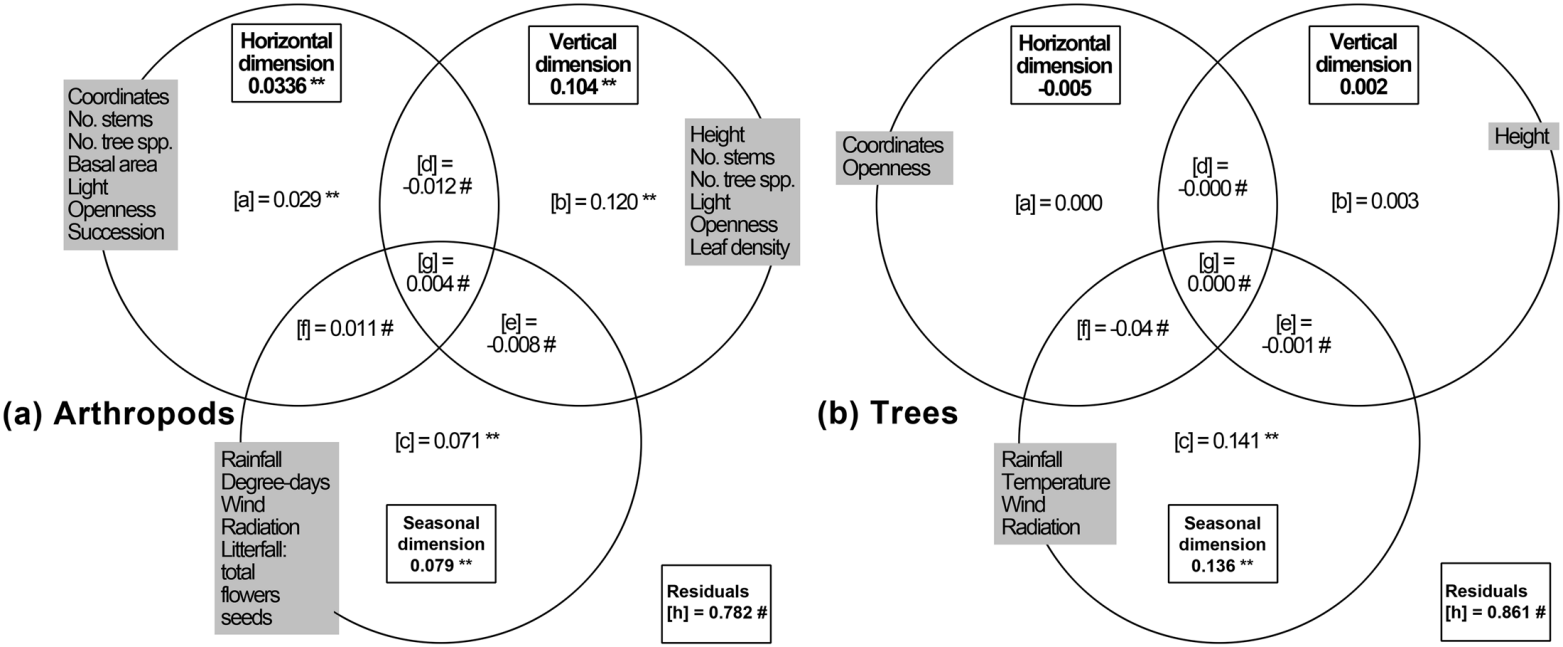
Venn diagrams summarizing canonical variation partitioning along three dimensions for (a) arthropods and (b) flowering trees. (a) 5858 arthropod species collected at 12 sites with all collecting methods. (b) 25 species of trees flowering during Surveys 1–4. Variables used to characterize the three dimensions are included in grey boxes. ** = significance of the fraction of variation (200 randomizations, p< 0.01); # fraction not testable. For description of fractions [a]-[h], see methods.

The variation in species composition explained by horizontal, vertical and seasonal variables was relatively low, between 15 and 28% for different data sets (22% for the data set with all species; Fig 5a and S7 Fig). Of the total fraction explained, however, measured variables consistently ordered the dimensions as vertical > seasonal > horizontal when considering the variation uniquely expressed by these dimensions (fractions [b], [c] and [a], respectively; ANOVA with arthropod guilds as data sets; F_2,12_ = 11.09, p < 0.01). The fraction of variation that was jointly explained by the different dimensions was rather low, with particularly strong effects of the vertical and seasonal dimensions alone (Fig 5a).

**Fig 6:**
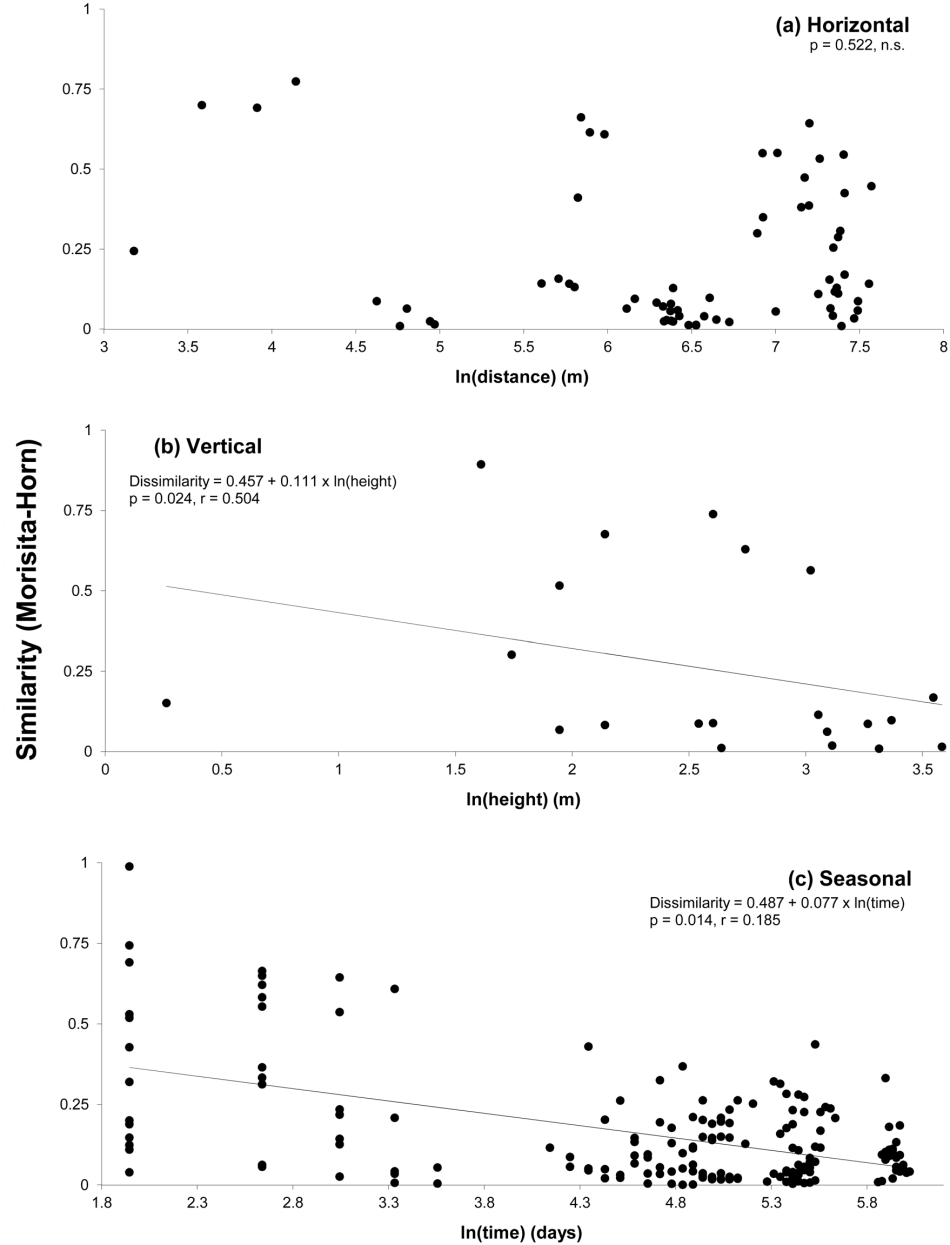
Arthropod species turnover, expressed by faunal similarity measured with the Morisita-Horn index, in the (a) horizontal, (b) vertical and (c) seasonal dimensions. Shown are the parameters of pairwise dissimilarity regressed on pairwise log(distance), with p values based on 1,000 permutations of pairwise distance versus pairwise dissimilarity matrices, and the overall concordance (r) between the matrices of observed and estimated values. Plotted models refer to the decay of similarity (i.e. 1-dissimilarity), for more intuitive interpretation.

We found no detectable gradient in the turnover of species composition in the horizontal dimension, with similarity sometimes remaining rather high after 1.5km (Fig 6a). However, in the vertical (Fig 6b) and seasonal dimensions, we detected statistically significant gradients (Fig 6c). Initial similarity was halved over 12m along the vertical gradient, and over 76 days along the seasonal gradient. Significant gradients of similarity existed mostly when considering habitats in the seasonal dimension (S8 Fig). Phytophages had a significant distance decay (halving of initial similarity after about 340 m), predators and fungal feeders with height, and ants and phytophages with time (S9 Fig).

### Comparison of arthropod vs. tree distribution

Effect sizes in the horizontal dimension for the variables of abundance, observed and estimated species richness were often larger for trees than for arthropods. In the vertical dimension, the outcome of these comparisons were not so clear, whereas in the seasonal dimension effect sizes were larger for arthropods than for trees (details in S2 Text). Out of 228 tree species occurring within our sites, 25 species were flowering at some point during Surveys 1–4. Additive decomposition of species richness across the horizontal, vertical and seasonal dimensions for flowering trees was not significantly different than that for arthropods, with α accounting for 12.8%, βT for 13.9%, βH for 27.6% and βV for 45.7% of turnover, respectively (G-test, G = 1.88, p = 0.60; Fig 4a). For flowering trees, sample size was too small to estimate alpha and the other multiplicative components of species diversity reliably. The variation explained in the species composition among samples of flowering trees by horizontal, vertical and seasonal variables was lower (14%) than for arthropods. Not surprisingly, this variation was mostly explained by seasonal variables, such as rainfall, temperature, wind and radiation (Fig 5b). There was a significant distance decay of similarity for all trees, but not for flowering trees (S10 Fig). Similarity for all tree species gradually declined to reach a value of 0.2 after 2km. All main results reported in this section are summarized in Table 2, with the strength of interactions estimated on the basis of effect sizes, R^2^ and p-values.

**Table 2:** Summary of the principal results, detailed for the horizontal, vertical and seasonal dimensions. 0 = no interaction, + = weak, ++ = intermediate, +++ = strong, NA = not available (see results), NT not tested (see methods). AR = arthropods, TR = trees.

Dimension / Analyses	All arthropods	Guilds	Trees
HORIZONTAL DIMENSION			
Abundance	+	0 for most	+, TR ≈ AR
Observed species richness	+	NT	++, TR > AR
Estimated species richness	+	NT	+++, TR > AR
Additive decomposition of species richness	++	++	++, TR ≈ AR
Multiplicative partitioning of species diversity	+	+++	NA
Variation in species composition	+	+	0, TR < AR
Turnover	0	+	+, TR > AR
VERTICAL DIMENSION			
Abundance	+	+++	+++, TR > AR
Observed species richness	+++	NT	+++, TR ≈ AR
Estimated species richness	+++	NT	+++, TR ≈ AR
Additive decomposition of species richness	+++	+++	+++, TR ≈ AR
Multiplicative partitioning of species diversity	+	+	NA
Variation in species composition	+++	+++	0, TR < AR
Turnover	++	++	NA
SEASONAL DIMENSION			
Abundance	+++	+++	++, TR < AR
Observed species richness	+++	NT	++, TR < AR
Estimated species richness	++	NT	+, TR < AR
Additive decomposition of species richness	+	+	+, TR ≈ AR
Multiplicative partitioning of species diversity	+++	+++	NA
Variation in species composition	++	++	+++, TR > AR
Turnover	++	++	++, TR ≈ AR

## Discussion

The spatio-temporal distribution of the most diverse group of eukaryotes on Earth, tropical rainforest arthropods, remains poorly understood. Thus, the basic documentation of patterns within even single forest constitutes a first step towards understanding how the main part of biodiversity is structured. In this study, we partitioned arthropod diversity and abundance into components of the horizontal (sites), vertical (habitats) and seasonal (surveys) dimensions of a tropical rainforest. Overall, the vertical and seasonal dimensions both had a stronger imprint on arthropod distribution than had the horizontal dimension (Table 2). In addition, arthropod guilds differed in their specific response to each dimension. Comparing arthropods to much-better studied trees, the horizontal dimension had a stronger impact on the distribution of trees than on arthropods, whereas the seasonal dimension had stronger effects on the distribution of arthropods than on trees. Below we discuss briefly salient questions related to the patterns observed along the horizontal, vertical and seasonal dimensions. We then turn to the main implications of our results for models of tropical beta diversity, global biodiversity estimates and the conservation of tropical arthropods.

With regard to the horizontal dimension, a meta-analysis by Soininen [12] indicated that a halving of the initial faunal similarity of a variety of assemblages occurred after 639 km (although few of these studies included arthropods). In our comparisons over distances of 24-1941m, no significant decay of similarity with distance was observed, with the exception of phytophages, for which initial similarity halved after 340 m (S9 Fig). Interestingly, several studies involving tropical moths [18,41] and to a lesser extent tropical insect herbivores [20] also reported a distance decay of similarity. Since moths are not that vagile and typically rather oligophagous, their distribution can be best thought of as paralleling that of their host plants, which in turn can be explained by neutral processes [18,27]. Our data indicate that this pattern is not shared with other arthropod guilds, which have a weaker association with plants than do the phytophages. While part of the current patterns could be biased towards high dissimilarities due to under-sampling bias (i.e. the effect of drawing a relatively small sample from a very large species pool), such biases should be similar in the three dimensions. Thus, comparisons of relative similarity should be valid among dimensions.

In which vertical habitat(s), then, do most arthropod species occur in the San Lorenzo forest? This is not an easy question to answer, because our protocols targeted adults whereas many larvae or nymphs may depend on habitats other than those where adults were collected (e.g. [42]). If we simply ask in which habitats do adult arthropod species concentrate, it is clear that a significant proportion of biodiversity occurs in the soil/litter (e.g. [16]), but that the rate of species accumulation in this habitat is not as steep as for the others (S4 Fig). The canopy is probably where a great many species thrive as adults (S2 Table, S4 and S5 Figs).

The seasonal dimension and associated variables (rainfall, degree-days, radiation, different categories of litterfall as proxy for plant phenology, Fig 5a) also had strong effects on the distribution of arthropods at San Lorenzo, as has been reported for many studies in tropical rainforests (reviewed in [15] and [43]). Our study included one full year of data and did not account for inter-annual variation in arthropod abundance and species richness, which may be pronounced in the tropics [17,23,24,44]. For example, important differences in seasonality exist among adult beetles of an Australian tropical rainforest depending on their feeding ecology, body size, and whether they live in the canopy or near the ground [44]. Studies of temperate and tropical beetles [44,45] also suggested that temporal patterns for arthropod species in temperate trees may not be more coordinated than those in tropical trees (i.e., tropical arthropods may also have well defined peaks in activity). A strong gradient of species turnover in the seasonal dimension as reported in this study is consistent with this contention. Our model further indicated that the initial faunal similarity of assemblages was halved in about 76 days (Fig 6). This represents a much shorter time than the average 226 days reported in the meta-analysis of Soininen [12].

Revisiting the main questions initially asked, we conclude the following:

1. The horizontal, vertical and seasonal dimensions all contribute significantly to the distribution in space and time of arthropods in the San Lorenzo forest. At the scale of our study (2km of distance, 40m in height and ca. 400 days of duration), effects related to the vertical and seasonal dimensions were equally important, followed by factors related to the horizontal dimension. We expect the effect of vertical factors to be even more significant at smaller scales, but to be progressively overshadowed by other factors at larger scales. Importantly, our results then indicate that in tropical lowland rainforests, factors related to the vertical dimension, which are often neglected, must be accounted for in any sound modeling of arthropod distribution in space and time. Our best models indicated that for the most disparate values recorded within this study in the horizontal (distance), vertical (height) and seasonal (time) dimensions, arthropod relative faunal similarity was 0.187, 0.116 and 0.108, respectively (average similarity = 0.256, 0.215 and 0.157, respectively). This suggests that arthropod assemblages are more similar in the horizontal dimension than in other dimensions, within the range of values targeted.
2. There were different patterns of distribution in space and time identifiable among arthropod guilds. In addition to the distance decay of similarity already discussed for phytophages, we can cite other examples. The abundance of ants was higher in the litter and canopy than in other habitats and significantly different among sites. This may reflect the sociality of these insects and requirements for the establishment of colonies. Phytophages were less abundant in the soil/litter as might be expected of primary consumers and, logically, a large fraction of their species richness was related to changes in the vertical dimension. These patterns were similar for predators, whose prey probably includes many phytophages. Fungivores were not so abundant in the soil/litter, whereas scavengers were abundant in this habitat but not so in the upper canopy. These observations emphasize the diversity in arthropod life-histories and the well known fact that it is near impossible to find examples of “umbrella species” for arthropods [46].
3. Based on our sampling design, we were able to make a direct comparison of patterns among trees and arthropods at a common scale. We believe that such comparisons are informative by highlighting the differential conservation needs of long- and short-lived organisms. For most responses assessed, effect sizes within the horizontal dimension were similar or larger for trees than for arthropods (Table 2). This observation probably relates to dispersal mode (and possibly to differing levels of under-sampling bias). This may be expected when comparing large and sedentary organisms dispersing with propagules (trees) vs. small and mobile organisms, most of them actively dispersing at the adult stage (arthropods). Within the vertical dimension, effect sizes were similar among trees and arthropods. This observation appears to be related to the distribution of juveniles and reproductive units. For trees, infertile saplings occur in the understory whereas most reproductive units occur in the canopy or upper canopy. For arthropods, immature stages frequently live in a different vertical habitat than do adults (often in the soil/litter vs. canopy/upper canopy, respectively) [15,16,42]. Finally, within the seasonal dimension, effect sizes were similar or smaller for trees than for arthropods. This observation is probably related to differences in the organisms’ lifespan. Long-lived organisms (trees) are more likely to be resilient in the face of seasonal weather extremes and buffered against shortages of resources than are short-lived organisms (arthropods).

Taken together, our findings come with three main implications. First, our data suggest that arthropods are more finely segregated along the seasonal dimension than are trees. Conversely, trees may be more finely segregated along the horizontal dimension than are arthropods. Given this contrast, serious questions must be asked whether models of beta diversity developed for tropical trees [27] are relevant to tropical arthropods, which represent the majority of biodiversity? For example, Condit et al. [27] examined a model based on the neutral theory [10] describing how tree similarity should change with distance in a community where only dispersal and speciation affect species distribution. The model fitted tree species distribution well from 0.2 to 50 km for datasets originating from Panama and Ecuador. We observed a distance decay of similarity in our tree data, but not in our arthropod data. Hence, it is unlikely that this ‘neutral’ tree model will be useful to predict the distribution of arthropods within the San Lorenzo forest. This is hardly surprising considering that trees and arthropods may represent two extremes of life-histories: large, sedentary and long-lived organisms vs. small, mobile and short-lived organisms, respectively. As discussed previously, phytophages may represent an exception to this rule because most species are strongly associated with their hosts. The one common denominator we identified for rainforest trees and arthropods is their dependence on factors related to the vertical dimension, particularly when considering reproductive units, and we believe that this area of research deserves more attention. Overall, our data suggest that tropical trees cannot necessarily be used as “umbrella species” [47] for arthropods. Previously, we showed that estimated arthropod species richness in the San Lorenzo forest could be well predicted from tree species richness but, consistent with this study, not so accurately from distance decay models [1]. Our current analyses suggest that these findings do not extend to the partitioning of arthropod diversity in space and time. While it may be possible to evaluate arthropod species richness from plant richness (and therefore implicitly to consider plants as umbrella species for arthropods in biodiversity hotspots [48]), equating the conservation needs of tropical arthropod species to those of tropical tree species by assuming similar distributional patterns seems an unjustified simplification.

Second, we have shown that arthropod species are particularly dependent on factors related to the vertical and seasonal dimensions. Unfortunately, these are also the dimensions which are the most difficult and costly to incorporate into protocols for surveying arthropods in tropical rainforests because they require extended sampling across seasons, as well as access to the vertical habitats available to arthropods in the canopy. Global biodiversity estimates rely heavily on the numbers of insect herbivores associated with tropical trees [2]. Our data suggest that sound estimates can only be attained if the original data include vertical and seasonal samples of insect herbivores. The latter are rarely adequate [49], but can be improved by stratified field protocols. In particular, vertical effects may be tempered by structured protocols allowing, for example, ground-level studies to catch larvae or emerging canopy-dwelling adults [42].

Last, the relatively high arthropod faunal similarity observed in the horizontal dimension suggests that dispersal limitation in this dimension is relatively weaker than within the vertical and seasonal dimensions, where we observed stronger decays in similarity with height or time. This has strong implications for the conservation of rainforest arthropods. Changes in the horizontal dimension may result mainly from the conversion of old-growth rainforests to secondary forests, plantations and other habitats (i.e., changes in variables such as number of stems and tree species, basal area and succession in our Fig 5a). Arthropod dispersal and fitness in these conditions may be impeded, but perhaps not as dramatically as thought, as evidenced by reports that converted forests still may sustain a high proportion of arthropod diversity [18,50]. However, changes in the vertical dimension may be promoted by similar changes in the horizontal dimension and, additionally, by any change in the canopy openness (variables light, openness, leaf density in Fig 5a) that may alter arthropod microhabitats along the vertical dimension, such as selective logging [18]. Drastic changes along the vertical profile, particularly where habitat continuity may be lost, such as in the canopy and upper canopy, may further limit arthropod dispersal and fitness, with canopy species being particularly at risk of local extinction. Changes in the seasonal dimension may be of special concern as dispersal limitation in this dimension may be related to the short lifespans of most adult arthropods. In this case, any changes in air temperature, rainfall, or resources driven by climate change (such as phenological mismatch with host plants or prey [51]), may strongly affect arthropod dispersal and fitness. Despite this, there are only a handful of long-term monitoring schemes have been dedicated to tropical arthropods [14,17,52]. We urgently call for the implementation of monitoring schemes to evaluate the effects of anthropogenic change on the spatio-temporal distribution of arthropod biodiversity, or we may grossly underestimate arthropod extinction risks in tropical rainforests.

## Supporting Information

S1 Fig Plot of canopy openness (best fit power relationship) versus arthropod abundance collected by sticky traps (780 traps, 3683 individuals, 9 sites, 4 habitats, 4 surveys). (TIF)

S2 Fig Mean abundance per sample detailed for each arthropod guild and site. For each guild, sites are plotted along the following sequences: B1, B2, C1, C2, C3, F1, F2, F3, I1, R1, R2 and R3. The p-values of ANOVAs for each guild are indicated. For the sake of clarity, s.e. are not plotted.
(TIF)

S3 Fig Mean abundance per sample detailed for each arthropod guild and survey. For each guild, survey are plotted along the following sequences: S1, S2, S3 and S4. The p-values of ANOVAs for each guild are indicated and different letters denote significantly different means (Tukey tests, p<0.05). For the sake of clarity, s.e. are not plotted.
(TIF)

S4 Fig Sample-based species accumulation curves plotted against a re-scaled axis of number of individuals sampled for (a) different sites, (b) different habitats and (c) different surveys. (TIF)

S5 Fig Representative box-plots of mean arthropod species richness across (a) sites, (b) habitats and (c) surveys. See Table 1 for details about data sets.
(TIF)

S6 Fig Multiplicative partitioning of species diversity for major data sets (all species collected with ten protocols, species collected with FITs, 885 common species) and major arthropod guilds. Plot of the multiplicative components of β: betaT (grey bars), betaH (stippled) and betaV (white). For rare species, parasitoids and flowering trees, sample sizes were too small to reliably estimate alpha and the other multiplicative components.
(TIF)

S7 Fig Percentage of total variation in species composition explained by variables accounting for the horizontal (stippled bars), vertical (white bars) and seasonal (grey bars) dimensions, for different data sets. Percentages refer to the fraction of variation uniquely explained by horizontal, vertical or seasonal variables. Entries above each data set indicate the total variation explained in the data set. ‘All spp.’ refers to the analysis detailed in Fig 3, for comparison with other data sets (5858 spp.). ‘4 sites’ refers to species collected with all methods at sites C1, C2, C3 and I1. ‘4 sites FIT’ refers to species collected at the preceding sites with intercept-flight traps only. All fractions are significantly non-random with p < 0.01 (200 randomizations). Variation partitioning analyses with rare species and parasitoids were not significant.
(TIF)

S8 Fig Plot of faunal similarity in (a) the horizontal dimension detailed for each habitat; (b) the horizontal dimension detailed for each survey; and (c) the seasonal dimension detailed for each habitat. Significant models of the form y = a + b ln(x) are also plotted (p<0.05, 1,000 permutations).
(TIF)

S9 Fig Plot of faunal similarity in (a) horizontal, (b) vertical and (c) seasonal dimensions, detailed for each arthropod guild. Significant models of the form y = a + b ln(x) are also plotted (p<0.05, 1,000 permutations).
(TIF)

S10 Fig Species turnover of trees (flowering trees in black, all trees in red), expressed by similarity measured with the Morisita-Horn index, in (a) horizontal and (b) seasonal dimensions. Shown are the parameters of pairwise dissimilarity regressed on pairwise log(distance), with p values based on 1,000 permutations of pairwise distance versus pairwise dissimilarity matrices, and the overall concordance (r) between the matrices of observed and estimated values. Plotted models refer to the decay of similarity (i.e. 1-dissimilarity), for more intuitive interpretation.
(TIF)

S1 Table Results of a mixed-effects ANOVA (habitats LIT, UND, CAN nested within sites C1, C2, C3, I1) with repeated measures (surveys 1, 2, 3, 4), with log arthropod abundance collected in FITs as the dependent variable (668 samples, 20,469 arthropods). (DOC)

S2 Table Results of Kruskall-Wallis tests (variable = median number of species collected per sample) comparing arthropod species richness among sites, habitats and surveys. Sobs = number of species observed; ES = effect size; Sest = number of species estimated by the Chao2 estimator. Datasets and codes of protocols as in Table 1. LIT = Litter, UND = Understory, CAN = Canopy, UPC = Upper canopy. Too few samples were available for a composite analysis of habitats.
(DOC)

S3 Table Results of a mixed-effects ANOVA (habitats LIT, UND, CAN nested within sites C1, C2, C3, I1) with repeated measures (surveys 1, 2, 3, 4), with log arthropod species richness collected in FITs as the dependent variable (668 samples, 20,469 arthropods). (DOC)

S1 Text Supplementary methods. (DOC)

S2 Text Supplementary results. (DOC)

## Abbreviations

DBH: diameter at breast height
FIT: flight-intercept trap
