# Supplementary figures and images for: Arthropod Distribution in a Tropical Rainforest: Tackling a Four Dimensional Puzzle
Source: PLoS One. 2015 Dec 3;10(12):e0144110. doi: 10.1371/journal.pone.0144110 (PMC4669110; doi:10.1371/journal.pone.0144110)

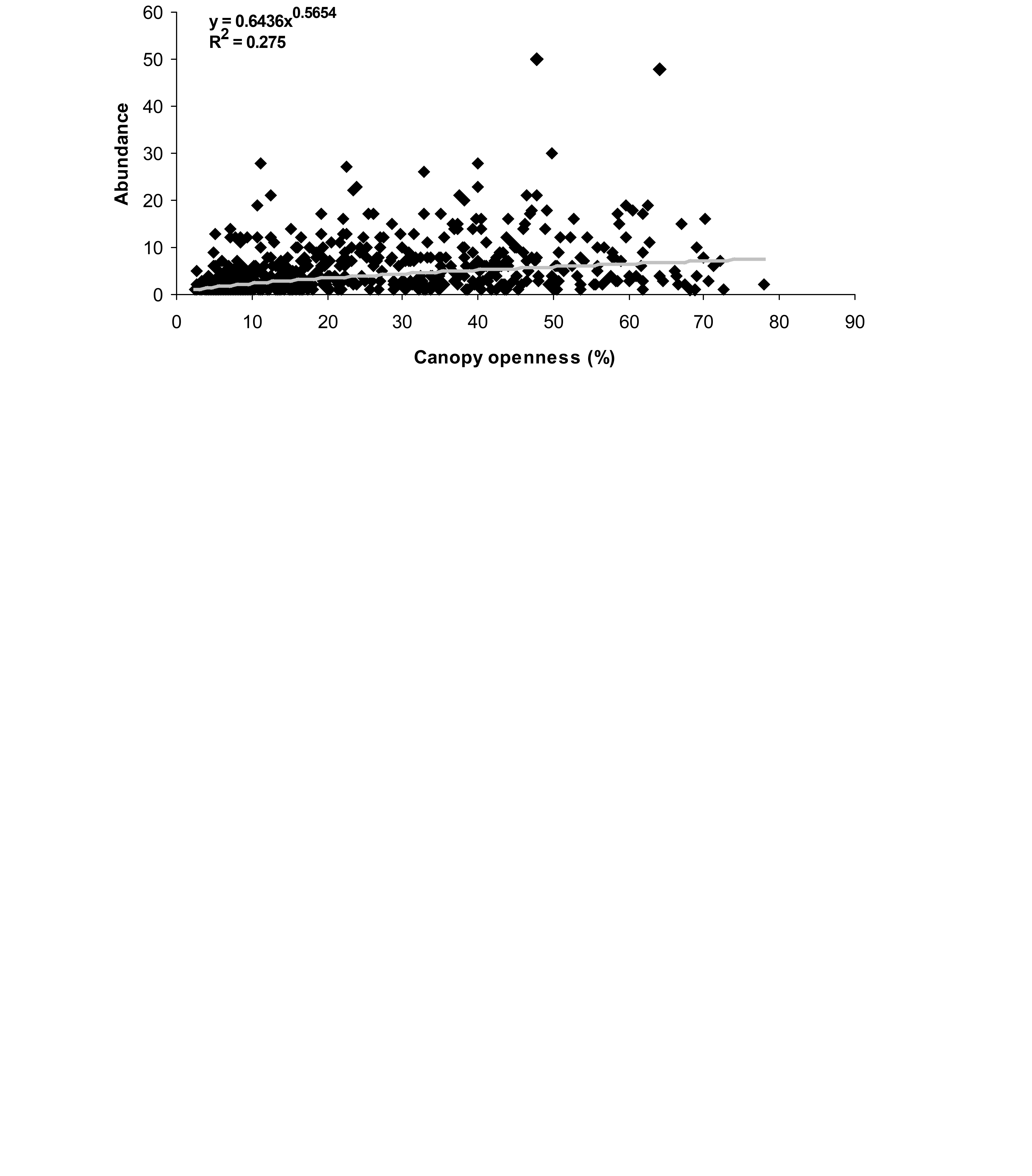

Supplement: S1 Fig — (TIF) [file pone.0144110.s001.tif]

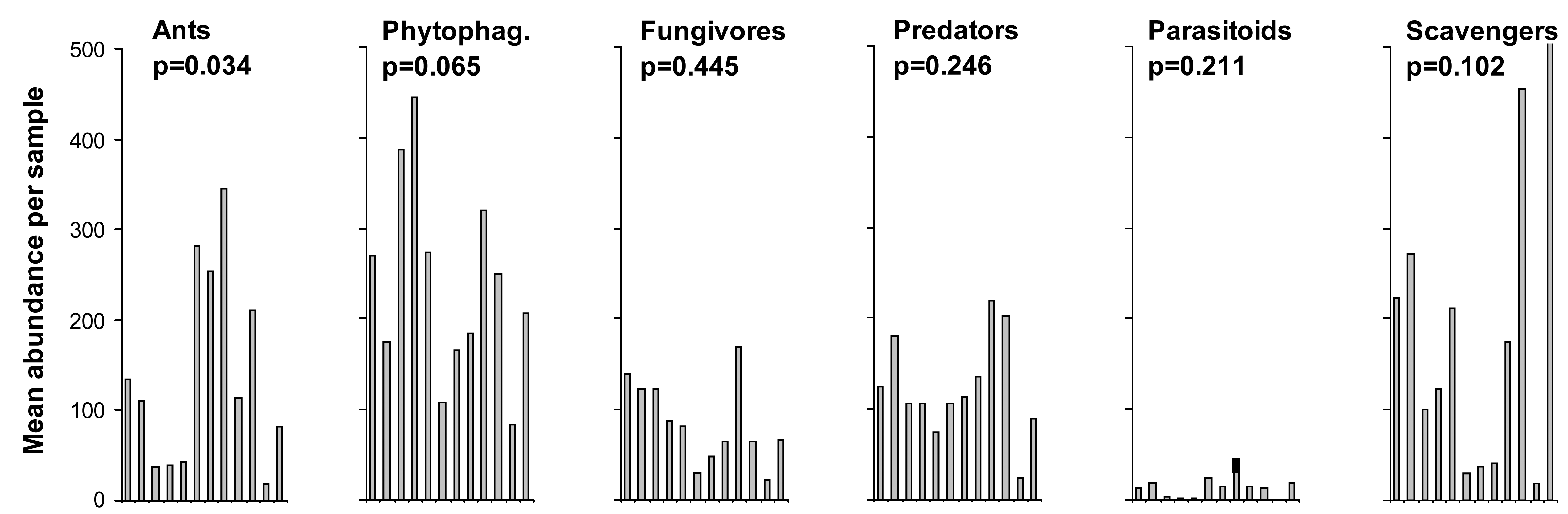

Supplement: S2 Fig — For each guild, sites are plotted along the following sequences: B1, B2, C1, C2, C3, F1, F2, F3, I1, R1, R2 and R3. The p-values of ANOVAs for each guild are indicated. For the sake of clarity, s.e. are not plotted. (TIF) [file pone.0144110.s002.tif]

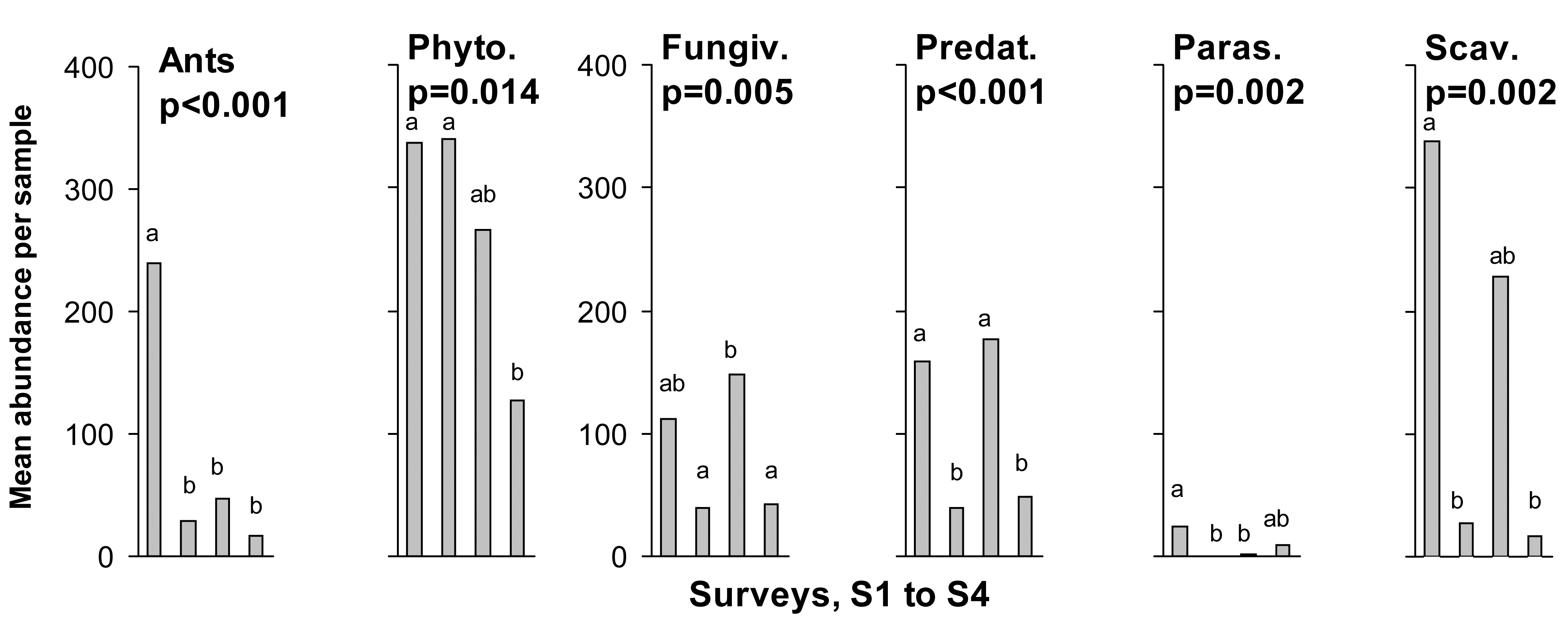

Supplement: S3 Fig — For each guild, survey are plotted along the following sequences: S1, S2, S3 and S4. The p-values of ANOVAs for each guild are indicated and different letters denote significantly different means (Tukey tests, p<0.05). For the sake of clarity, s.e. are not plotted. (TIF) [file pone.0144110.s003.tif]

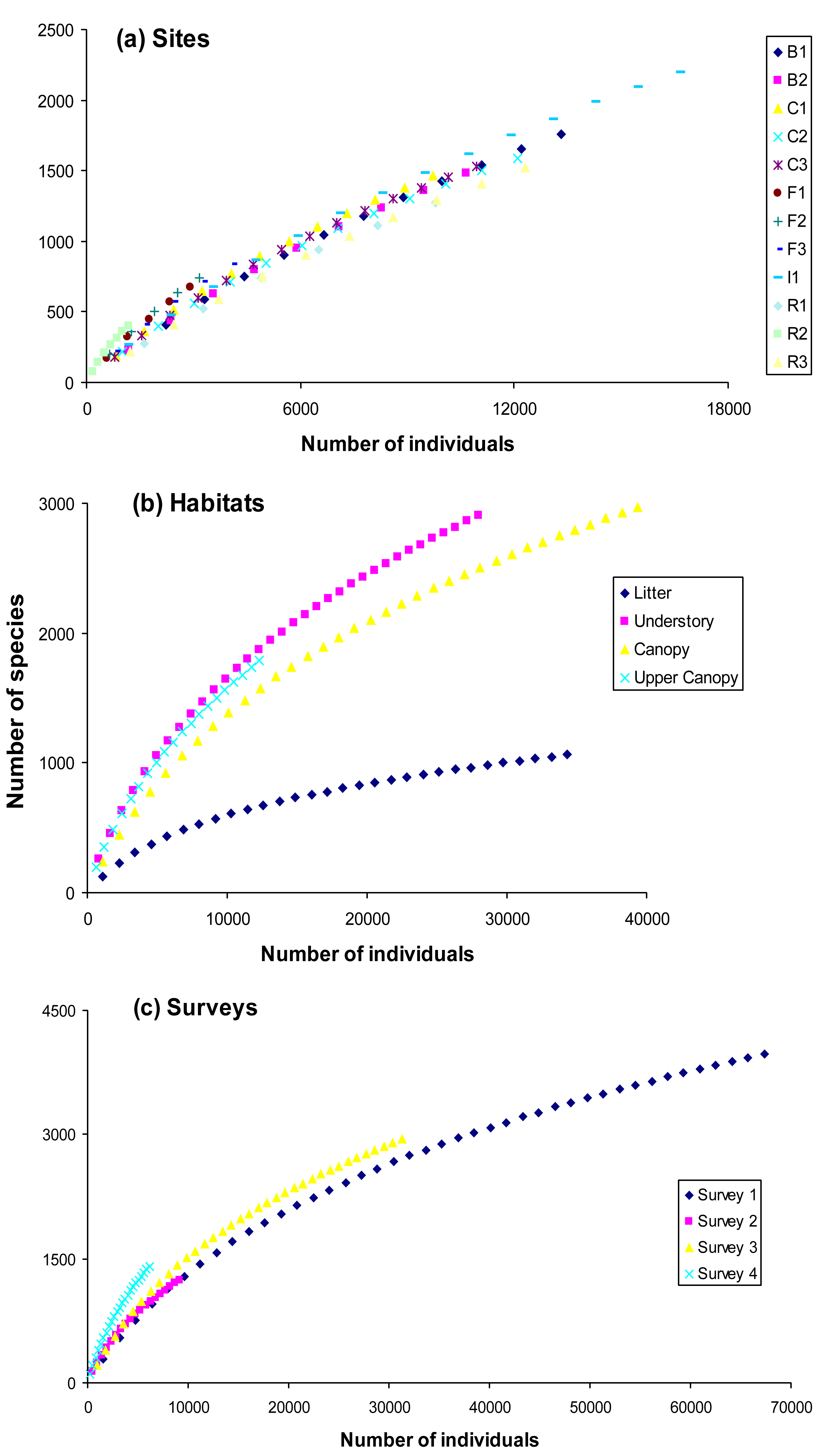

Supplement: S4 Fig — (TIF) [file pone.0144110.s004.tif]

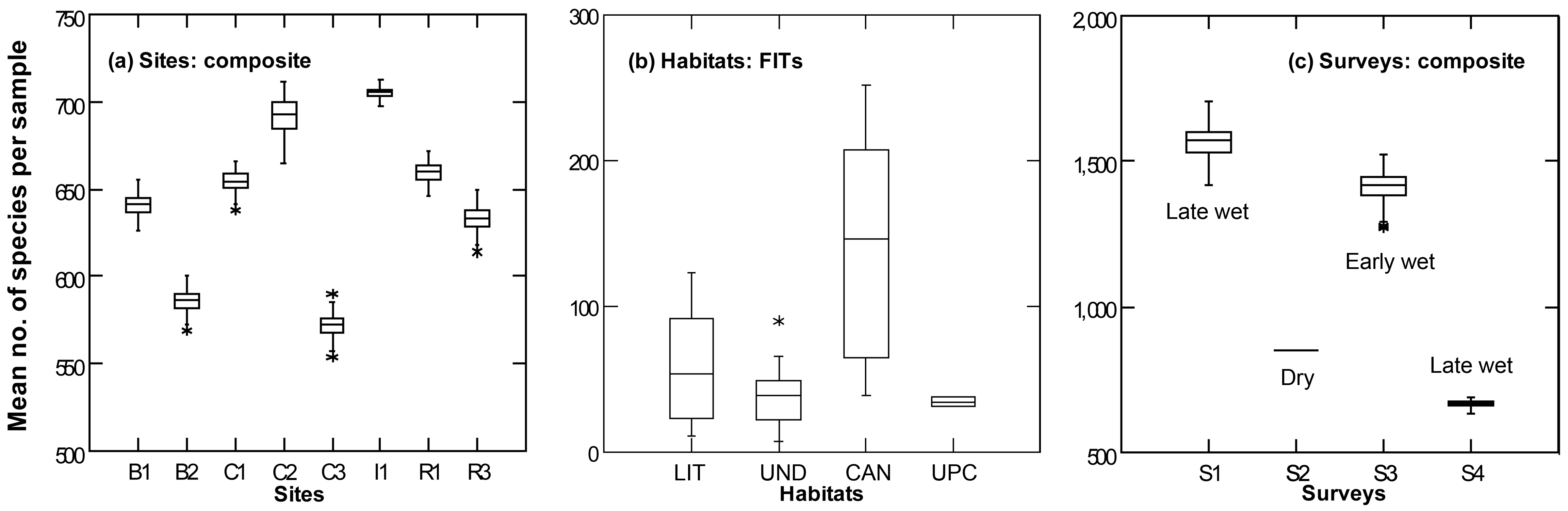

Supplement: S5 Fig — See Table 1 for details about data sets. (TIF) [file pone.0144110.s005.tif]

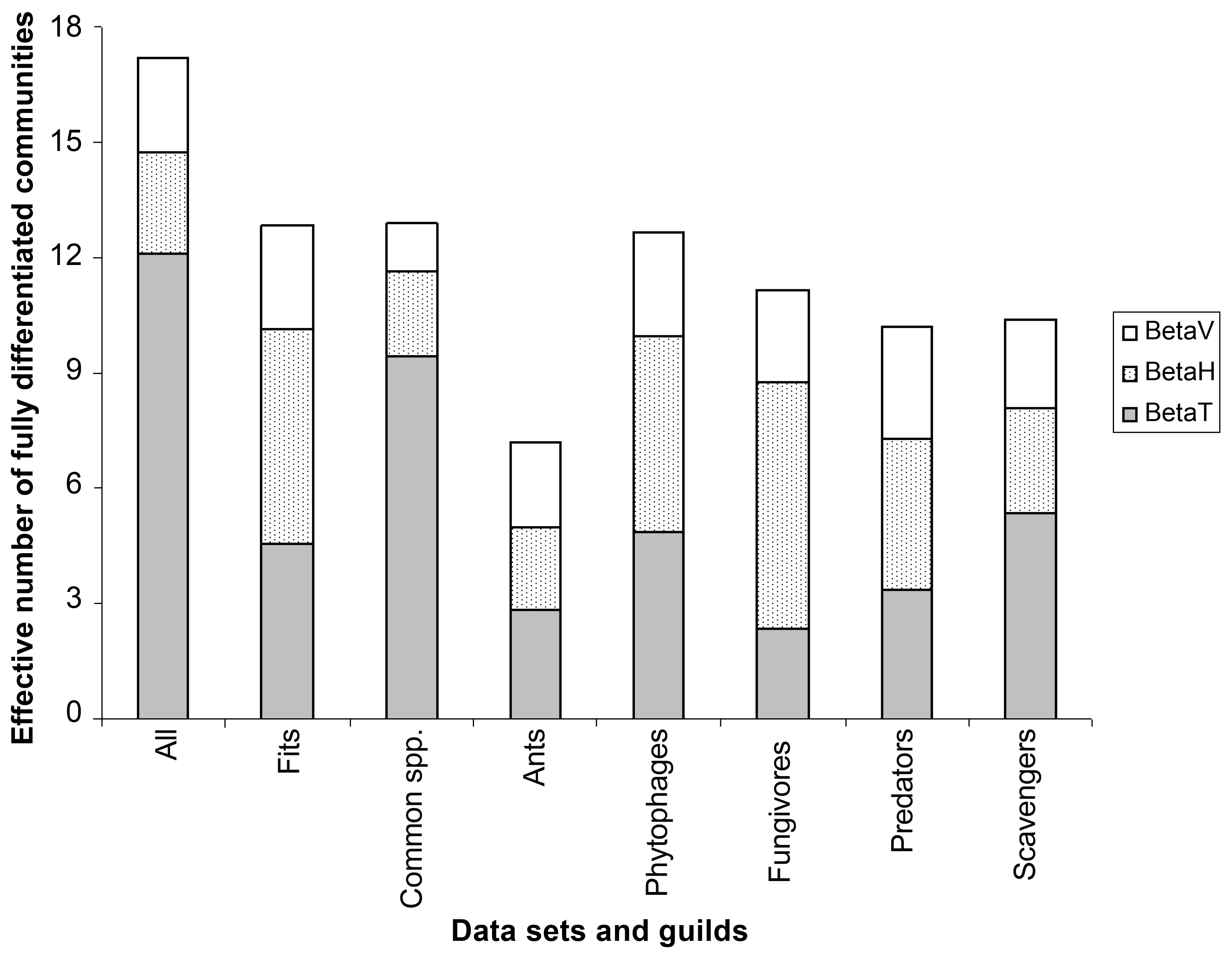

Supplement: S6 Fig — Plot of the multiplicative components of β: betaT (grey bars), betaH (stippled) and betaV (white). For rare species, parasitoids and flowering trees, sample sizes were too small to reliably estimate alpha and the other multiplicative components. (TIF) [file pone.0144110.s006.tif]

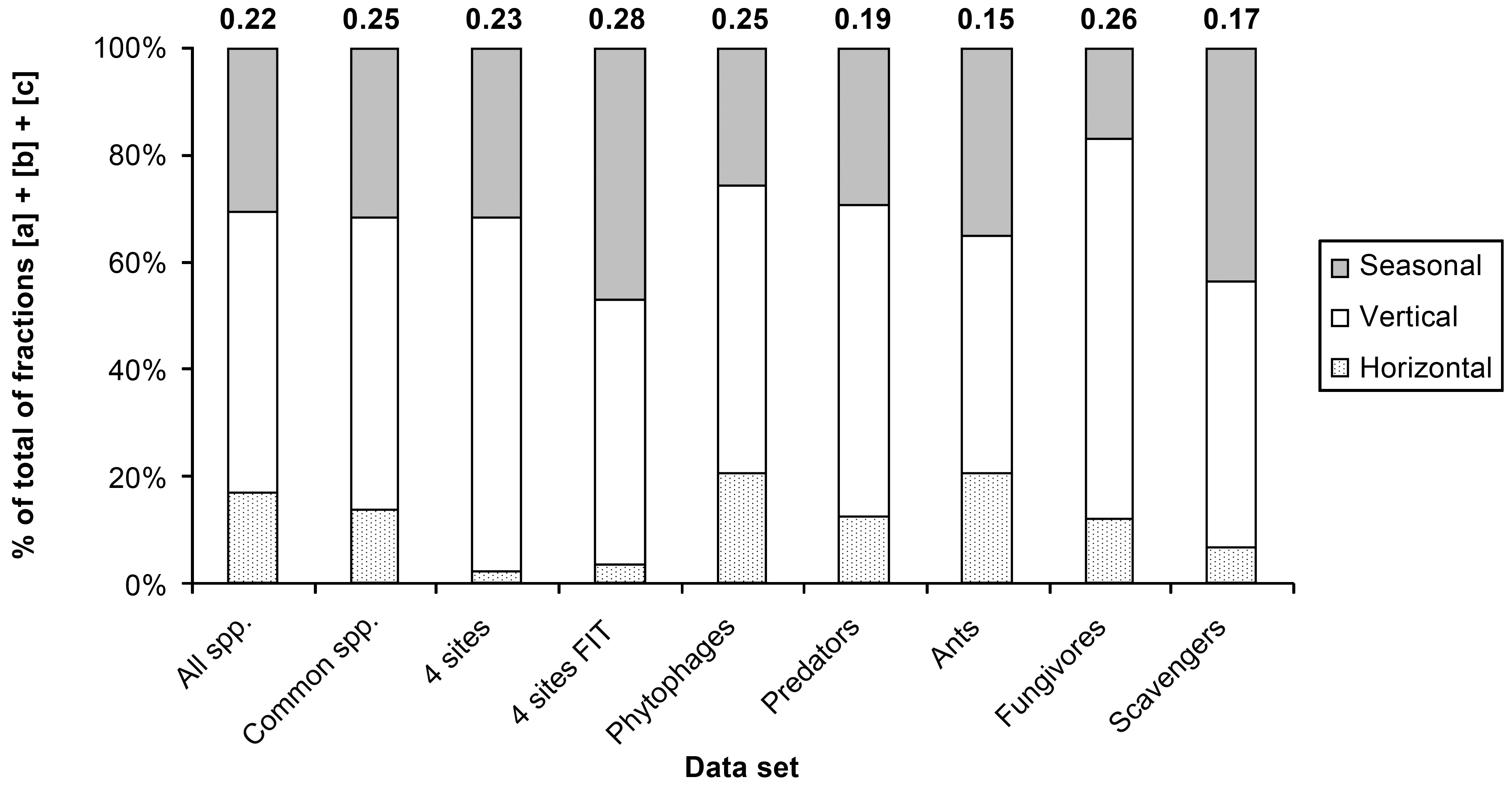

Supplement: S7 Fig — Percentages refer to the fraction of variation uniquely explained by horizontal, vertical or seasonal variables. Entries above each data set indicate the total variation explained in the data set. ‘All spp.’ refers to the analysis detailed in Fig 3, for comparison with other data sets (5858 spp.). ‘4 sites’ refers to species collected with all methods at sites C1, C2, C3 and I1. ‘4 sites FIT’ refers to species collected at the preceding sites with intercept-flight traps only. All fractions are significantly non-random with p < 0.01 (200 randomizations). Variation partitioning analyses with rare species and parasitoids were not significant. (TIF) [file pone.0144110.s007.tif]

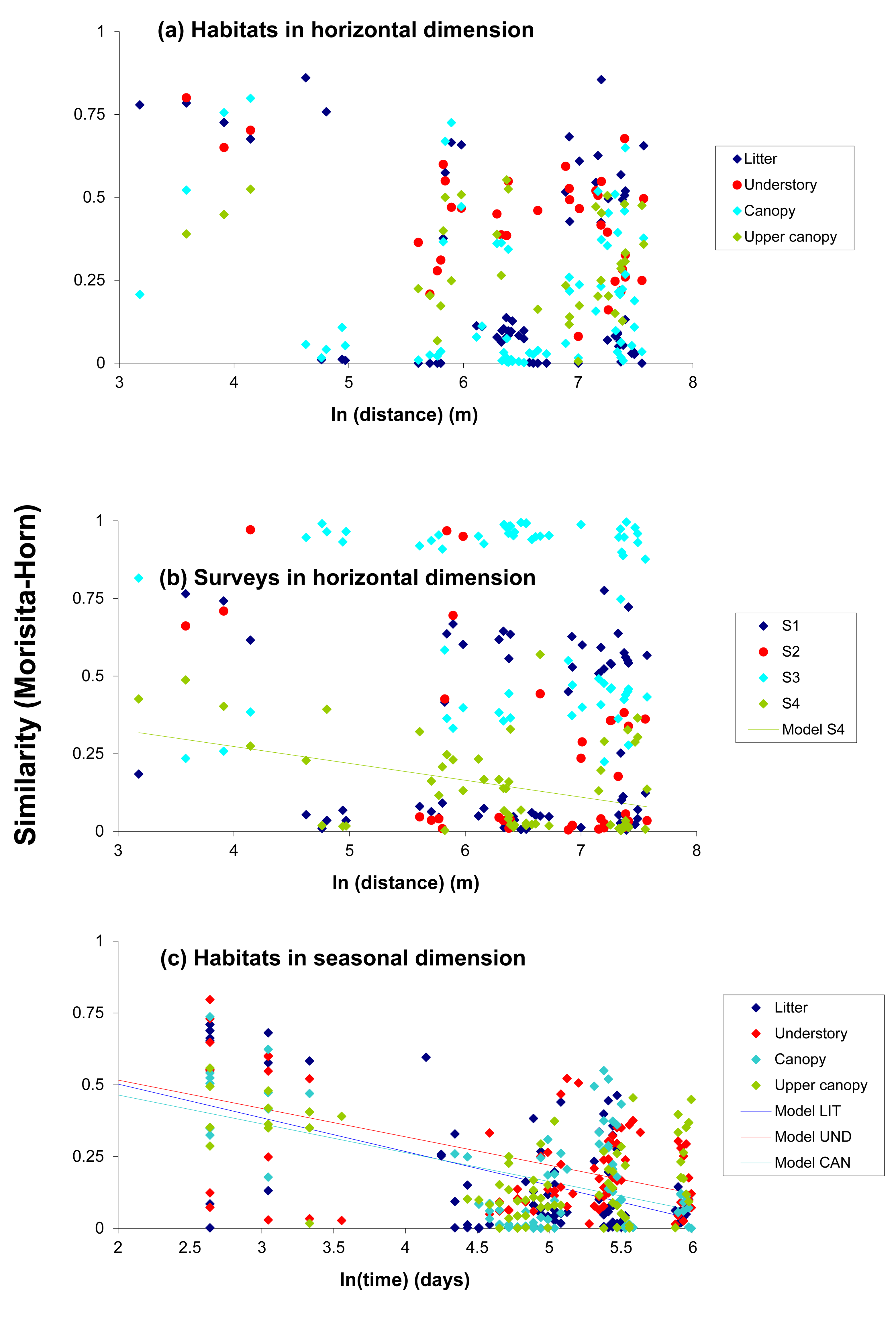

Supplement: S8 Fig — Significant models of the form y = a + b ln(x) are also plotted (p<0.05, 1,000 permutations). (TIF) [file pone.0144110.s008.tif]

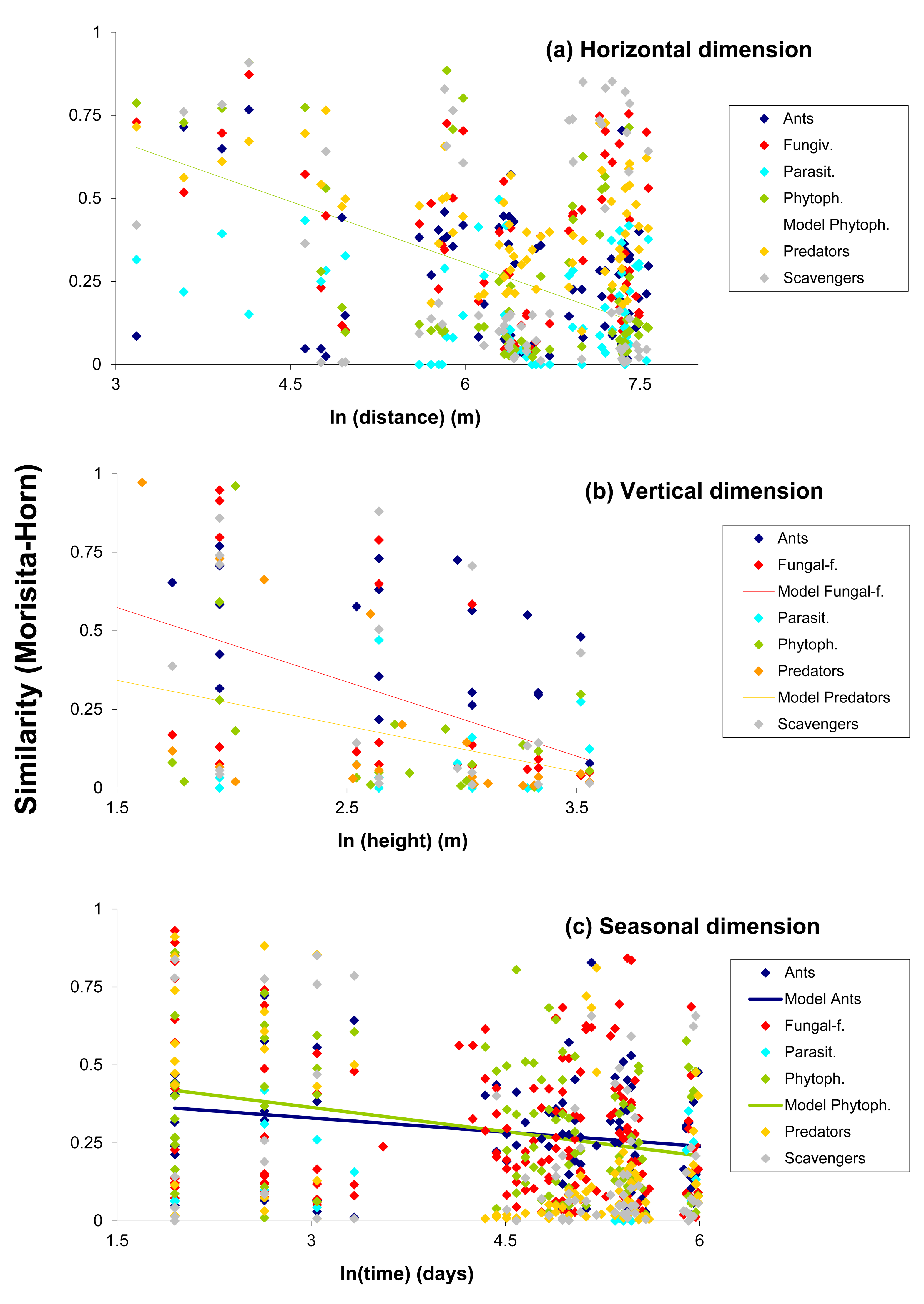

Supplement: S9 Fig — Significant models of the form y = a + b ln(x) are also plotted (p<0.05, 1,000 permutations). (TIF) [file pone.0144110.s009.tif]

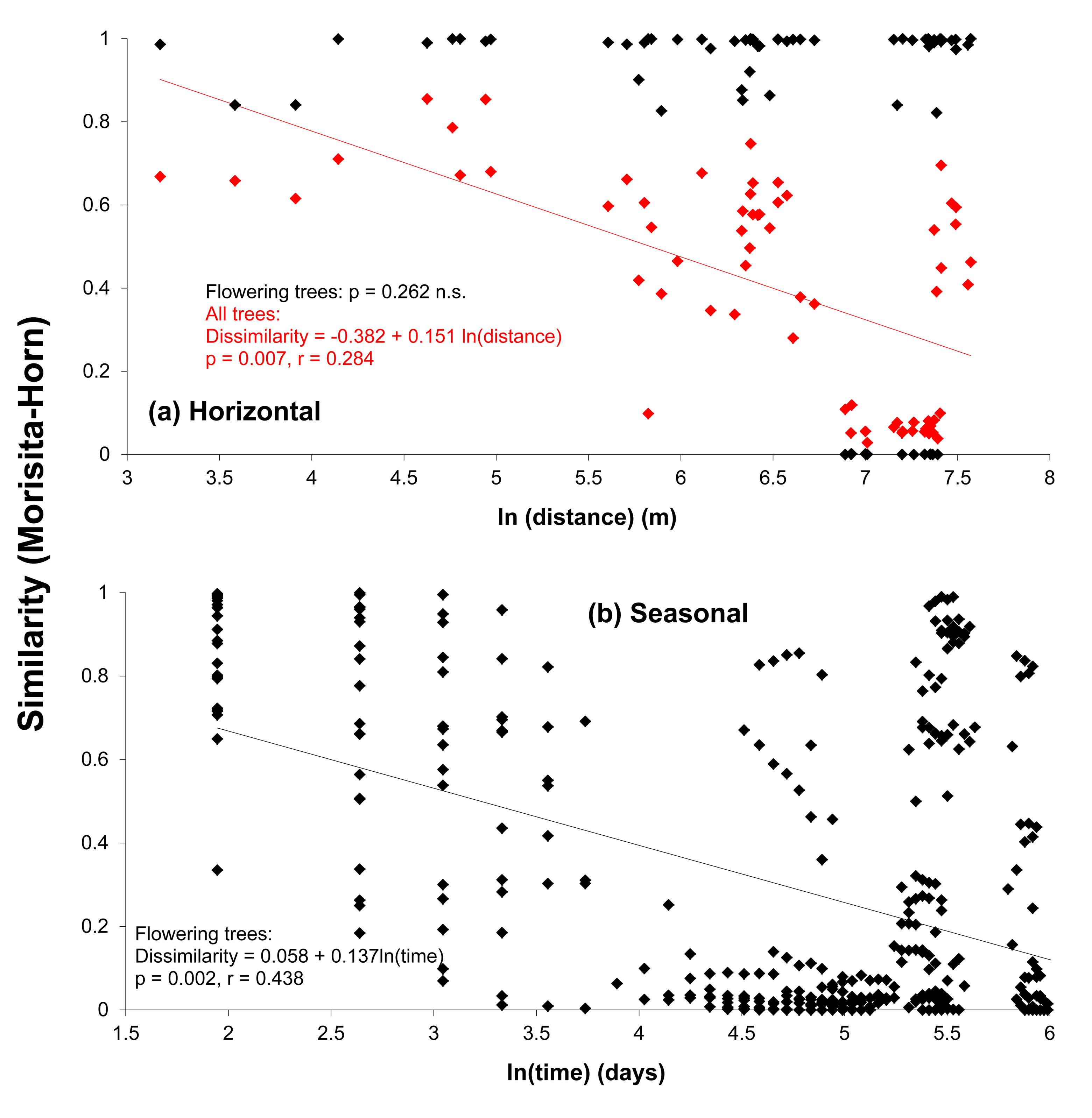

Supplement: S10 Fig — Shown are the parameters of pairwise dissimilarity regressed on pairwise log(distance), with p values based on 1,000 permutations of pairwise distance versus pairwise dissimilarity matrices, and the overall concordance (r) between the matrices of observed and estimated values. Plotted models refer to the decay of similarity (i.e. 1-dissimilarity), for more intuitive interpretation. (TIF) [file pone.0144110.s010.tif]
