# Supplementary material for: Arthropod Distribution in a Tropical Rainforest: Tackling a Four Dimensional Puzzle
Source: PLoS One. 2015 Dec 3;10(12):e0144110. doi: 10.1371/journal.pone.0144110 (PMC4669110; doi:10.1371/journal.pone.0144110)
Supplement: S1 Table — (DOC) [file pone.0144110.s011.doc]

**S1 Table.** Results of a mixed-effects ANOVA (habitats LIT, UND, CAN nested within sites C1, C2, C3, I1) with repeated measures (surveys 1, 2, 3, 4), with log arthropod abundance collected in FITs as the dependent variable (668 samples, 20,469 arthropods).

| **Source** | | Sum-of-Squares | **df** | Mean-of-Squares | F | ***p*** |
| --- | --- | --- | --- | --- | --- | --- |
|  | Between samples |  |  |  |  |  |
|  | Site | 2.588 | 3 | 0.863 | 3.736 | 0.013 |
|  | Habitat(Site) | 2.970 | 8 | 0.371 | 1.608 | 0.131 |
|  | Error | 25.171 | 109 | 0.231 |  |  |
|  | Within samples |  |  |  |  |  |
|  | Survey | 25.990 | 3 | 8.663 | 47.520 | 0.000 |
|  | Survey*Site | 1.393 | 9 | 0.155 | 0.849 | 0.571 |
|  | Survey*Habitat(Site) | 3.427 | 24 | 0.143 | 0.783 | 0.758 |
|  | Error | 59.614 | 327 | 0.182 |  |  |
