# Supplementary material for: Arthropod Distribution in a Tropical Rainforest: Tackling a Four Dimensional Puzzle
Source: PLoS One. 2015 Dec 3;10(12):e0144110. doi: 10.1371/journal.pone.0144110 (PMC4669110; doi:10.1371/journal.pone.0144110)
Supplement: S2 Table — Sobs = number of species observed; ES = effect size; Sest = number of species estimated by the Chao2 estimator. Datasets and codes of protocols as in Table 1. LIT = Litter, UND = Understory, CAN = Canopy, UPC = Upper canopy. Too few samples were available for a composite analysis of habitats. (DOC) [file pone.0144110.s012.doc]

**S2 Table.** Results of Kruskall-Wallis tests (variable = median number of species collected per sample) comparing arthropod species richness among sites, habitats and surveys. Sobs = number of species observed; ES = effect size; Sest = number of species estimated by the Chao2 estimator. Datasets and codes of protocols as in Table 1. LIT = Litter, UND = Understory, CAN = Canopy, UPC = Upper canopy. Too few samples were available for a composite analysis of habitats.

| **Analysis** | **Sobs** |  |  |  | **Median no. spp.** | |  |  |  | **Sest±SD** |  |  |  | **KW / *p*** |
| --- | --- | --- | --- | --- | --- | --- | --- | --- | --- | --- | --- | --- | --- | --- |
|  | **Min.** | **Max.** | **ES** |  | **Min.** | **Max.** | **ES** |  | **Min.** | **Max.** | **ES** |  |  |
|  | **(state)** | **(state)** |  |  | **(state)** | **(state)** |  |  | **(state)** | **(state)** |  |  |  |
| Sites: STIs | 47(R1) | 97(R3) | 0.347 |  | 1(*) | 2(*) | 0.333 |  | 126±25(B2) | 237±79(C1) | 0.306 |  | 34.0 / <0.001 |
| Sites: FOG | 216(R3) | 357(F3) | 0.246 |  | 44(R3) | 91(I1) | 0.348 |  | 559±59(B1) | 800±82(F3) | 0.177 |  | 18.1 / 0.011 |
| Sites: FITs | 241(C3) | 367(B1) | 0.207 |  | 10(C3) | 16(B1) | 0.231 |  | 505±62 (C3) | 925±109(B1) | 0.294 |  | 34.7 / <0.001 |
| Sites: composite | 606(B2) | 725(C2) | 0.089 |  | 572(C3) | 705(I1) | 0.104 |  | 1192±51(B2) | 1811±82(C2) | 0.206 |  | 762.8 / <0.001 |
| Habitats: STIs | 34(LIT) | 307(UPC) | 0.801 |  | 3(LIT) | 40(UPC) | 0.860 |  | 134±58(LIT) | 683±80(UPC) | 0.671 |  | 46.9 / <0.001 |
| Habitats: FITs | 56(UPC) | 1132(CAN) | 0.906 |  | 35(UPC) | 146(CAN) | 0.618 |  | 88±14(UPC) | 2415±143(CAN) | 0.929 |  | 21.2 / <0.001 |
| Surveys: FITs | 264(S2) | S3(863) | 0.531 |  | 20(S4) | 99(S1) | 0.664 |  | 543±62(S2) | 1847±125(S3) | 0.546 |  | 27.1 / <0.001 |
| Surveys: LITs | 317(S4) | 1461(S1) | 0.643 |  | 42(S4) | 78(S1) | 0.303 |  | 675±72(S4) | 2551±106(S1) | 0.581 |  | 20.5 / <0.001 |
| Surveys: PITs | 22(S2) | 159(S1) | 0.757 |  | 2(S2) | 5(S1) | 0.429 |  | 35±8(S4) | 344±53(S1) | 0.816 |  | 26.1 / <0.001 |
| Surveys: composite | 795(S2) | 1572(S1) | 0.328 |  | 667(S4) | 1570(S1) | 0.404 |  | 1523±49(S4) | 3659±184(S1) | 0.412 |  | 378.0 / <0.001 |
