# Supplementary material for: Arthropod Distribution in a Tropical Rainforest: Tackling a Four Dimensional Puzzle
Source: PLoS One. 2015 Dec 3;10(12):e0144110. doi: 10.1371/journal.pone.0144110 (PMC4669110; doi:10.1371/journal.pone.0144110)
Supplement: S1 Text — (DOC) [file pone.0144110.s014.doc]

**S1 Text. Supplementary methods.**

**Taxonomic analyses and guild assignment.** Focal arthropods were sorted to named species or morphospecies by taxonomists (see [1] for a list of focal taxa and taxonomists). We previously estimated that our focal taxa represented about 45% of all arthropod species collected at San Lorenzo [1]. Admittedly, this is far from representing an exhaustive survey of the arthropod fauna of San Lorenzo, but this is nevertheless more comprehensive than most of the tropical arthropod studies conducted to date (review in [53]). Arthropods were assigned to feeding guilds [54]. Guild assignment for beetles was challenging since many species can be assigned into categories that mix up feeding relations and structural niches [55]. Hence, we followed different authors [56-58] to assign beetle species to a modified guild system (detailed in [1]). The main arthropod guilds eventually retained for our analyses included ants, phytophages (including chewers and sap-suckers), fungivores, predators, parasitoids and scavengers. Note that only a small proportion of parasitoid species were sorted and this included mostly Braconidae. Guild structure included number of individuals instead of number of species as using species is less robust due to potential influx of transient species [59].

**Arthropod abundance, observed and estimated species richness.** The flight-intercept trap protocol allowed a full factorial analysis for a restricted number of sites. Further we also considered “composite” analyses that included a higher proportion of arthropods collected. For these analyses, the results of several protocols were pooled, with the prerequisite that the number of samples was identical for each site, habitat or survey considered (details in Table 1). To achieve this, datasets were randomized 100 times using EstimateS 8.20 [36] and we considered the results for the lowest common number of samples obtained at each site or survey (details in Table 1). A mixed-effects ANOVA model considering habitats nested within sites was performed with repeated measures (surveys) and the log-transformed number of individuals or species collected in each sample as a dependent variable.

**Test for differences in species accumulation curves.** Sample-based species accumulation curves were re-scaled to number of individuals collected on the x-axis. Accumulation curves across different sites, among different habitats and among different surveys fitted the Weibull function well (all R^2^ > 0.99). Because the Weibull function is non-linear, an analysis of the residual sum of squares [60] was used to test whether a single Weibull curve could be fitted to all groups compared, using the following F-statistic: ((SScombined – SSseparate) / (DFcombined – DFseparate)) / (SSseparate / DFseparate), where SS is the sum-of-squares and DF the degrees of freedom.

**Randomization methods to test for differences in species richness among samples.** We tested the null hypothesis that communities were drawn from the same underlying population (same regional species pool), irrespective of sample size, and that individuals occurred at random and with equal probability in every sample (Richardson & Richards, 2008). To test for differences among groups, we estimated how likely it was that the observed data would occur if individuals from the common species pool were distributed at random among groups [61]. This required an estimate of the distribution of species-richness values among sites under the assumption of random placement. To generate this distribution under the null model, we pooled all individuals of every species into one large group and then randomly re-assigned individuals to samples, with the constraint that total abundance in each sample and for each species remained fixed at observed values [62]. Once all individuals had been randomly re-assigned to a sample, species richness was calculated for each sample and also for each group. This generated a set of ‘pseudo-values’ of species richness under the null hypothesis. The whole procedure was repeated 10,000 times to generate a frequency distribution of expected species richness per sample and per group. The observed values of species richness could then be compared to those expected under the null hypothesis, and if the observed species richness was less than the lowest 2.5% or greater than the highest 2.5% of the randomization estimates, we concluded that the underlying species pools differed significantly [61,62]. Richardson and Richards [62] provide a convenient program to perform the test and JML Richardson kindly recompiled a version to accept 6,000 species.

**Additive decomposition** **of species richness.** Common and rare species were defined as the first and last quartile of a species abundance distribution (3,541 species in total, 885 species for each category) and representing 90% and 2% of total abundance (47,485 individuals), respectively. Partitioning of species richness other than for common and rare species was performed with all species available, including singletons. Most of the additive decomposition of species richness was performed with PARTITION [63], which includes an individual-based randomization procedure where the statistical significance of level-specific alpha and beta estimates can be tested. Individuals from a single species-abundance distribution were randomly assigned (without replacement) to samples such that the number of individuals within the sample was maintained. This was repeated 1,000 times and the data were partitioned so as to obtain a null distribution of each alpha and beta estimate at each level of analysis. Statistical significance was assessed by the proportion of null values that were greater (or less) than the actual estimates. The technique does not require rarefaction of the data prior to analyses, so differences in sampling effort among replicates within a given level do not bias partitions [64]. We also described α and βT as linear regressions of several independent variables measured at sites, within habitats or during surveys (see methods). In these multiple regressions, we included log arthropod abundance to reduce the importance of sample size [65].

**Multiplicative partitioning of species diversity**. It was calculated as follows. We first used a script written in R [66] to calculate, for each sample, Hurlbert’s effective number of species (ENS; [32]). The ENS is defined as the number of equi-frequent species that would lead to the same diversity index [32,33,67]. Following Dauby and Hardy [35], the number of species in each sample was rarefied to a common sample size, as to calculate ENS for *k* individuals. This procedure yields a measure of diversity that gives the highest possible weight to rare species without bias [32]. ENS values were then substituted for the number of species to calculate the different components of diversity as γ = α × βT × βH × βV [32,33], in a fashion similar to that used for the additive decomposition of species richness (see above). Note that in this case the multiplicative components of β can be interpreted as the effective number of fully differentiated communities [32]. Briefly stated, additive decomposition of species richness accounts for the change in species richness irrespective of their relative abundance, whereas multiplicative partitioning of species diversity accounts for the effective number of fully differentiated communities (i.e., compositional dissimilarity), taking into account the relative commonness and rarity of species.

**Beta diversity.** Concepts and analyses related to beta diversity are numerous and often present contrasting approaches with subtle distinctions [37]. Anderson *et al*. [37] provided a timely review of these concepts and we adopt their terminology. They distinguish two types of beta diversity: turnover (directional) and variation (non-directional). Turnover measures the change in community structure from one sampling unit to another along a spatial, temporal or environmental gradient. Variation in community structure is measured among a set of sample units within a given spatial or temporal extent. It is measured among all possible pairs of units, without reference to any particular gradient or direction [37]. Our horizontal dimension of beta diversity measured among sites in the forest is not necessarily directional, and considers only a spatially-implicit distance-decay of faunal similarities among sites. Our vertical dimension of beta diversity measured from the soil to the upper canopy could be considered as a directional gradient but habitats along this gradient may be discrete, as for example the discontinuity between the soil and other habitats. Our temporal dimension of community change may be viewed as neither being directional or non-directional but rather cyclical, as recent long-term data on tropical butterflies have indicated [17]. Hence, both of the concepts of variation and turnover may be variously appropriate to our data sets and may help to interpret our results. The former concept may be the most appropriate for our data set as we have previously noted that distance-decay models did not explain the accumulation of arthropod species particularly well in the San Lorenzo forest as compared to models based on plant species richness [1].

**Variation in species composition.** We used the Hellinger distance, which is a dissimilarity coefficient that is quantitative, excludes joint absences and is appropriate for beta diversity analyses [40]. We partitioned variation in community composition among sample units defined as a combination of sites, habitats and surveys, with a series of seven canonical redundancy analyses (RDA) using the R-language function varpart in the vegan library [68]. For this analysis we retained the 5,858 species collected with all sampling methods at the 12 sites. To appraise the effect of sample size (number of individuals and species collected per sample), we also considered the more restricted data sets including species collected with all methods at four sites (C1, C2, C3 and I1), and species collected with FITs at four sites. We further ran similar analyses of variation partitioning for common and rare species and also for the main arthropod guilds. Three groups of explanatory variables (see methods) were used to represent variation in the horizontal, vertical and seasonal dimensions in the variation partitioning analyses. Using these variables, variation in arthropod species composition was divided into eight fractions: (a) variation uniquely explained by geographical distances and horizontal variables; (b) variation uniquely explained by vertical variables; (c) variation uniquely explained by seasonal variables; (d) variation jointly explained by horizontal and vertical variables; (e) variation jointly explained by vertical and seasonal variables; (f) variation jointly explained by horizontal and seasonal variables; (g) variation jointly explained by all three groups of variables; and (h) residual unexplained variation. Where possible, the significance of fractions was tested by RDA models with 200 permutations. However, not all fractions can be tested since the number of fractions is higher than the number of estimated models and non-testable fractions frequently yield negative estimates of variation [68].

**Species turnover.** As a measure of similarity, we used the Morisita-Horn index as calculated in the vegan library of R [68]. Assuming a constant decay from an initially higher level of similarity (S) towards a lower level, we fitted a negative exponential function to data on all pairwise similarities versus pairwise distance in the horizontal, vertical or temporal dimension, respectively. Parameter estimation was implemented by fitting linear similarity versus log-transformed distance (d) in program ISOLDE in GENEPOP v. 4.2 R [69,70], thus obtaining estimates of parameters a and b in S=be^ad^. The significance of the fit was evaluated by comparing the observed Spearman rank correlation r_s_ to a frequency distribution generated by 1,000 random permutations of the similarity versus log-distance matrices (Mantel test implemented in ISOLDE). A measure of concordance between the matrices of observed and fitted values was provided by a further Mantel test and its r statistics (1,000 permutations). We also contrasted faunal similarity in each habitat along the horizontal dimension, in each season along the horizontal dimension, and in each habitat along the seasonal dimension.

**Explanatory variables.** To quantify horizontal gradients, we considered the number of woody stems (DBH ≥10 mm), the number of woody plant species, basal area, reflected light (lux; average from 28 measurements within each site, measured with a digital lux meter LX-1010B; Kaito Electronics Inc., Montclair, CA 91763, USA; cell facing down at breast height), canopy openness (%; proportion of visible sky open to closed, measured with Hemiview 2.0 software from hemispherical pictures), and an index of succession [71] based on floristic composition. All of these variables are detailed in [30]. Instead of using geographic coordinates as variables belonging to the horizontal dimension in canonical variation partitioning, we used the principal coordinates of a matrix of geographic neighbors among the sampling sites (PCNM). The advantage of PCNM is that spatial dependence can be detected over a wider range of scales [72]. PCNM variables were extracted from grid transect data using the program SpaceMaker2 [73].

With respect to vertical gradients, we used an allometric equation relating DBH to height in order to assign individual plants within sites to either of the four habitats, and considered the number of stems and the number of tree species present. Tree height was measured using a laser range finder at the San Lorenzo crane site (as in [74] and used to derive an equation relating tree height to tree diameter at breast height across other sites (Height(m) = 0.7911xDBH(mm)^0.5553, R^2^ = 0.96, n= 66; S.A. Bohlman & R. Condit, unpubl. data). Thus, the foliage of individuals with DBH ≤12mm is likely to be mostly available for insects in the understory, whereas the foliage of individuals with DBH ≥250mm is likely to be mostly available for insects in the upper canopy. Plants with 12mm > DBH < 250mm are more likely to have their foliage in the canopy. The soil/litter habitat was assigned 0 for the number of stems and tree species. We also considered the height of the trap, the average reflected light (lux) and crown openness per habitat (%; both measured at the location of each sticky trap deployed), and leaf density for each site and habitat [75].

Seasonal gradients were characterized by the sum of rainfall (mm; sum of rainfall for year 2003 = 3478 mm, for year 2004 = 2696mm; sum of rainfall during dry season in 2003 = 254mm, in 2004 = 308mm); the sum of degree-days (ºC); the average wind speed (km/h); the sum of solar radiation of daily totals (moles × m^-2^); and the sum of average weekly litterfall (g dry weight). The first four variables were derived from meteorological data collected at the San Lorenzo canopy crane (<http://www.stri.si.edu/sites/esp//description_fts.htm>). Degree-days represent a better predictor than maximum air temperature for arthropods and were calculated using the rectangle method [76], where:

Degree-days = ((Maximum temperature + Minimum temperature)/2) - Minimum threshold,

with Minimum threshold set to 18º C (i.e., minimum temperature during the night in the tropics). Litterfall was estimated using weekly censuses of 40 litter traps located in the botanical plot (0.25m^2^ each, S.J. Wright, unpubl. data 1998–2005; methods as in [77]). We considered litterfall dry weight as the total material collected in traps, and the leaves, flowers and seeds, separately. The sum or average of these seasonal variables was considered over the 30 days preceding the mid-point of the period of sampling for each site and survey, as this time lag has been shown to be relevant to tropical arthropods (e.g., [78,79]). A few samples had missing values for particular variables. In order to avoid losing information, we replaced missing values by the average value of the measured variable in the dataset [80].

# Additional references

53. Basset Y (2001) Invertebrates in the canopy of tropical rain forests: how much do we really know? Plant Ecol 153: 87-107.

54. Moran CV, Southwood TRE (1982) The guild composition of arthropod communities in trees. J Anim Ecol 51: 289-306.

55. Schmidl J, Bussler H (2008) Xylobiontic beetle guild composition and diversity driven by forest canopy structure and management. In: Floren A, Schmidl J, editors. Canopy arthropod research in Europe. Nuremberg: Bioform Entomology. pp. 299–323.

56. Hammond PM (1990) Insect abundance and diversity in the Dumoga-Bone National Park, N. Sulawesi, with special reference to the beetle fauna of lowland rain forest in the Toraut region. In: Knight WJ, Holloway JD, editors. Insects and the rain forests of South East Asia (Wallacea). London: Royal Entomological Society of London. pp. 197–254.

57. Beutel RG. Leschen RAB, editors. (2005) Handbook of Zoology, part 38, Coleoptera, Beetles, vol. 1. Berlin: De Gruyter.

58. Leschen RAB, Beutel RG, Lawrence JF, editors. (2010) Handbook of Zoology, part 39, Coleoptera, Beetles, vol. 2. Berlin: De Gruyter.

59. Basset Y, Samuelson GA, Miller SE (1996) Similarities and contrasts in the local insect faunas associated with ten forest tree species of New Guinea. Pac Sci 50: 157-183.

60. Ratkowsky DA (1983) Nonlinear regression modelling: a unified practical approach. New York: Dekker.

61. Manly BFJ (1997) Randomization, bootstrap and Monte Carlo methods in biology, 2nd edition. , Boca Raton, Florida: Chapman & Hall.

62. Richardson JML, Richards MH (2008) A randomisation program to compare species-richness values. Ins Conserv Div 1: 135-141.

63. Veech JA. Crist TO (2009) PARTITION: software for hierarchical partitioning of species diversity, version 3.0. <http://www.users.muohio.edu/cristto/partition.htm>.

64. Crist TO, Veech JA, Gering JC, Summerville KS (2003) Partitioning species diversity across landscapes and regions: a hierarchical analysis of α, β, and γ diversity. Am Nat 162: 734-743.

65. Magurran AE (2004) Measuring biological diversity. Oxford: Blackwell.

66. R Core Team (2014) R: A Language and Environment for Statistical Computing. Vienna: R Foundation for Statistical Computing. [http://www.R-project.org](http://www.R-project.org/).

67. Chase JM, Knight, TM (2013) Scale-dependent effect sizes of ecological drivers on biodiversity: why standardised sampling is not enough. Ecol Lett 16: 17-26.

68. Oksanen J, Guillaume Blanchet F, Kindt R, Legendre P, O'Hara RB, Simpson GL et al. (2011) vegan: Community Ecology Package. R package version 1.17-6. Vienna: R Foundation for Statistical Computing. <http://CRAN.R-project.org/package=vegan>

69. Raymond M, Rousset F (1995) GENEPOP (version 1.2): population genetics software for exact tests and ecumenicism. J. Heredity 86: 248-249. Available at <http://genepop.curtin.edu.au/genepop_op6.html>

70. Rousset F (2008) Genepop'007: a complete reimplementation of the Genepop software for Windows and Linux. Mol. Ecol. Resources 8: 103-106.

71. Welden CW, Hewett SW, Hubbell SP, Foster RB (1991) Sapling survival, growth and recruitment: relationship to canopy height in a Neotropical forest. Ecology 72: 35-50.

72. Borcard D, Legendre P (2002) All-scale analysis of ecological data by means of principal coordinates of neighbour matrices. Ecol Modell 153: 51-68.

73. Borcard D, Legendre P (2004) SpaceMaker2 – User’s guide. Département de sciences biologiques, Université de Montréal. 20 pages. Available from <http://www.fas.umontreal.ca/biol/legendre/>.

74. Bohlman SA, O’Brien S (2006) Allometry, adult stature and regeneration requirement of 65 tree species on Barro Colorado Island, Panama. J Trop Ecol 22: 123–136.

75. Ribeiro SP, Basset Y (2007) Gall-forming and free-feeding herbivory along vertical gradients in a lowland tropical rainforest: the importance of leaf sclerophylly. Ecography 30: 663-672.

76. Arnold CY (1960) Maximum and minimum temperatures as a basis for computing heat units. Proc Am Soc Hortic Sci 74: 430–445.

77. Wright SJ, Calderón O, Hernandéz A, Paton S (2004) Are lianas increasing in importance in tropical forests? A 17-year record from Panama. Ecology 85: 484-489.

78. Thaiutsa B, Granger O (1979) Climate and the decomposition rate of tropical forest litter. Unasylva 126: 28-38.

79. Medianero E, Castaño-Meneses G, Tishechkin A, Basset Y, Barrios H, Ødegaard F et al. (2007) Influence of local illumination and plant composition on the spatial and seasonal distribution of litter-dwelling arthropods in a tropical forest. Pedobiologia 51: 131-145.

80. Lepš J, Šmilauer P (2003) Multivariate analysis of ecological data using CANOCO. Cambridge: Cambridge University Press.
