# Supplementary material for: Arthropod Distribution in a Tropical Rainforest: Tackling a Four Dimensional Puzzle
Source: PLoS One. 2015 Dec 3;10(12):e0144110. doi: 10.1371/journal.pone.0144110 (PMC4669110; doi:10.1371/journal.pone.0144110)
Supplement: S2 Text — (DOC) [file pone.0144110.s015.doc]

**S2 Text. Supplementary results.**

**Arthropod abundance.** Analyses suggested that arthropod abundance was lower at sites C1, C2 and C3 than at other sites; higher in the upper canopy than in other habitats; and lower during Survey 4 than during other surveys (Fig. 2). There was a weak positive correlation (<30% of variance explained) between arthropod abundance collected by sticky traps and both the canopy openness above the trap (Fig. S1) and the height of the trap (not presented, R^2^=0.29).

#### **Arthropod species richness and diversity.** Despite a very high total sampling effort, a complete census of arthropod species richness at San Lorenzo was not achieved (S4 Fig.). Although the steepness of species accumulation curves did not appear to be visually very different among sites (S4 Fig.), curves were significantly different (analysis of the residual sum of squares, F_44,63_ = 8485.5, p <0.0001). Differences were more acute between surveys, with a steeper accumulation of species during Survey 4 (F_12,103_ = 10,411, p <0.0001), as well as between habitats, with the litter sampling accumulating fewer species than in other habitats (F_12,103_ = 3.1 x 10^6^, p <0.0001; S4 Fig.). The randomization program (with 10 000 iterations) indicated that the null hypothesis of no difference in species richness between sites could be rejected, as well as that of no difference in species richness between habitats or between surveys (all with p <0.05).

Effect size was significantly different among factors (habitat, survey and site, in decreasing order) for the analysis reported in Table S2 when considering estimated species richness (Kruskal-Wallis test, W = 7.28, p < 0.05), but not so when considering the median number of species collected per sample (Kruskal-Wallis test, W = 5.65, p = 0.059). In addition, the factorial analysis on the number of species collected per FIT sample indicated that the factor survey was significant whereas the other factors were not (S3 Table). Sites B2 and C3 were lowest in species richness and, as for arthropod abundance, Survey S4 was also lowest in number of species collected compared with other surveys. There were discrepant results between the sticky and FITs with regards to habitats. Both analyses agree that the mean number of species collected per sample was higher in the canopy than in the litter. The highest mean number of species, however, was collected in the upper canopy for sticky traps (data not presented), and in the canopy for FITs (S5 Fig.). As for arthropod abundance, there was a positive relationship between either percentage canopy openness above the trap (r_s_ = 0.514, p <0.001, n = 707) or trap height (r_s_ = 0.497, p <0.001, n = 756) and the number of arthropod species collected with sticky traps.

**Additive decomposition** **of species richness.** Proportions of each beta diversity component were similar between species collected by FITs and species collected by the ten protocols combined (Chi-square = 1.80, p = 0.613), indicating that our scaling scheme from FITs to all protocols conserved the patterns observed for the FIT protocol. However, the additive decomposition for the number of species *estimated* with the ten protocols indicated a proportionally greater vertical turnover than for the number of o*bserved* species collected with the ten protocols (Chi-square = 20.01, p < 0.001).

The alpha diversity of species collected by the FITs could be explained with a significant multiple regression including the log arthropod abundance (standardized regression coefficient: 0.747) and total litterfall material (-0.346; R^2^ = 0.74, F_2,45_= 66.2, p <0.001). Similarly, the alpha diversity of species collected with all methods could be explained by litterfall- flower fraction only (0.442) and total litterfall material (-0.398; R^2^ = 0.41, F_2,45_= 17.4, p <0.001). The βT of species collected in FITs could be explained by a regression including the height of the trap (standardized regression coefficient: 0.762), log arthropod abundance (0.422), and leaf density (-0.201; R^2^ = 0.99, F_3,8_= 297.2, p <0.001). However, it was not possible to explain similarly the βT of species collected with all methods.

**Variation in species composition.** Variation partitioning for common species was very similar to that for the whole data set (G = 1.02, p = 0.59; S6 Fig.). However, the analysis was not significant for rare species or for the parasitoid guild (randomization tests on particular fractions were not significant). Not surprisingly, data sets including only four of the 12 sites had a significantly lower fraction of explained variation expressed in the horizontal dimension (comparison of all species with species collected at four sites using all methods: G = 37.1, p < 0.001; S7 Fig.). Across arthropod guilds, the fraction of variation explained in the vertical dimension was unusually high for the species composition of fungivores (G = 20.1, p < 0.001), while the fraction of variation explained in the seasonal dimension was unusually high for the species composition of scavengers (G = 13.3, p < 0.01).

**Species turnover.** There was no significant similarity decay with distance for each of the habitats (litter, undestory, canopy and upper canopy) considered separately (S8a Fig.). Similarly similarity decay with distance was not consistent across the four surveys, with only a significant model for the S4 data (S8b Fig.). However, all habitats but upper canopy showed a significant similarity decay with time, emphasizing the strong influence of the seasonal dimension (S8c Fig.). The slopes of significant models for litter, understory and canopy were fairly similar (S8c Fig.). Phytophagous arthropods were the only guild for which a significant model of similarity decay with distance existed (S9a Fig.). In this case, initial similarity was halved after about 340 m of distance. Within the vertical dimension, significant models of similarity decay with height existed for fungal feeders and predators, with a steeper decay slope for the former (S9b Fig.). Within the seasonal dimension significant models of similarity decay with time existed for ants and phytophagous arthropods, with fairly similar slopes (S9c Fig.).

#### **Comparison of arthropod vs. tree distribution.** (i) Abundance, observed and estimated species richness - The effect size (ES) in the horizontal dimension was similar for numbers of tree stems and arthropod abundance (ES for trees = 0.340; for arthropods average ES of 0.355 for data in Table 1). In the vertical dimension, the effect size was much larger for trees (ES= 0.905) than for arthropods (average ES = 0.391). In the seasonal dimension, the effect size was larger for arthropods than for trees (average ES = 0.546 for arthropods, ES = 0.458 for trees). With regard to observed or estimated species richness, the effect size was larger in the horizontal dimension for trees (ES = 0.352 and ES = 0.773, respectively) than it was for arthropods (average ES in Table S2 = 0.222 and ES = 0.245, respectively). In the vertical dimension, the effect size for observed and estimated species richness was similar for trees (ES = 0.805 and ES = 0.589, respectively) and for arthropods (average ES = 0.853 and ES = 0.800, respectively). In the seasonal dimension, the effect size for observed and estimated species richness was larger for arthropods (average ES = 0.564 and 0.589, respectively) than for trees (ES = 0.394 and ES = 0.394, respectively).

*(ii) Species turnover -* Overall, average pairwise similarity in the horizontal dimension was significantly different between taxa, with arthropods (0.215±0.027 [s.e.]) < all trees species (0.408±0.032) < tree species flowering (0.808±0.045; ANOVA, F_2,195_ = 72.0, p <0.0001, Tukey-tests all significant with p<0.05). We could not estimate the rate of floral turnover in the vertical dimension with the data at hand due to lack of vertical replicates and data limitations in the litter/understory (DBH of measured trees ≥ 10 cm). However it is probably considerable, as evidenced by the average low similarity between the understory, canopy and upper canopy (similarity of trees species flowering = 0.005). In the seasonal dimension, average pairwise similarity was also significantly lower for arthropods (0.157±0.013) than for flowering trees (0.305±0.018; t-test, t = 5.20, df = 547, p <0.001). A similarity of about 0.2 was reached after 60 days for arthropods whereas a similarity of 0.2 was reached after 245 days for tree species flowering (compare S6c Fig. and S10b Fig.).
